# Hybrid Photoelectrodes Based on Electropolymerized Conjugated Porous Polymers for Enhanced Solar Energy Conversion

**DOI:** 10.1002/smsc.202400623

**Published:** 2025-02-20

**Authors:** Elena Alfonso‐González, Miguel Gomez‐Mendoza, Carmen G. López‐Calixto, Miguel García‐Tecedor, Ignacio J. Villar‐García, Freddy Oropeza, Marta Liras, Mariam Barawi Moran, Víctor A. de la Peña O´Shea

**Affiliations:** ^1^ Photoactivated Processes Unit IMDEA Energy Institute Avda. Ramón de la Sagra 3 28935 Móstoles Madrid Spain; ^2^ Institute of Ceramics and Glass (ICV) CSIC Campus de Cantoblanco UAM‐CSIC, Kelsen 5 28049 Madrid Spain; ^3^ Department of International Security Peace Research Institute Frankfurt (PRIF) Baseler Strasse 27‐31 60329 Frankfurt am Main Germany; ^4^ Department of Chemistry Faculty of Pharmacy San Pablo CEU University Montepríncipe Urbanization 28668 Boadilla del Monte Madrid Spain

**Keywords:** conjugated porous polymers, electrochemical impedance spectroscopies, hybrid photoelectrodes, photoelectrochemistries, solar energy conversions, transient absorption spectroscopies

## Abstract

This work highlights and offers fundamental insights on the potential of electropolymerized conjugated porous polymers in developing efficient hybrid photoelectrodes for photoelectrochemical applications. A simple and cost‐effective electropolymerization strategy to create hybrid organic–inorganic photoelectrodes based on two thiophene‐based conjugated porous polymers (CPP‐3TB and IEP‐19) for enhanced solar energy conversion is used. These polymers, when integrated with TiO_2_ to form hybrid photoanodes, exhibit enhanced photopotentials and photocurrents compared to bare TiO_2_. This synergetic behavior is attributed to an increased visible light absorption, reduced charge transfer resistance, and minimized electron–hole recombination. In particular, detailed electrochemical and spectroscopic analyses, including electrochemical impedance spectroscopy and transient absorption spectroscopy, reveal that the hybrid systems’ superior charge transport and longer photogenerated charge lifetimes contribute to their increased efficiency in solar energy conversion. Moreover, by comparing the structure and behavior of both hybrid systems, corner stone knowledge for the synthesis of CPPs to guide the construction of the future photoelectrochemical cells for solar energy conversion is offered.

## Graphical abstract

This study investigates electropolymerized organic polymers and hybrids for solar energy conversion. The hybrids demonstrate enhanced photocurrents and charge transfers. The associated mechanisms are explored by advanced spectroscopies, highlighting the superior performance of the hybrids in general and CPP‐3TB in particular. A cost‐effective and reproducible synthesis is demonstrated, paving the way for their use in photoelectrochemical applications.

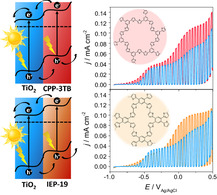

## 1 Introduction

In the pursuit of sustainable energy solutions, solar energy conversion has emerged as a pivotal field with the potential to mitigate our growing energy demands while reducing the carbon footprint. Central to the development of efficient solar energy conversion systems is the design and optimization of photoelectrodes, which play a critical role in the conversion of sunlight into usable electrical energy.^[^ 1, 2 ^]^ Photoelectrochemical (PEC) cells hold the potential to efficiently convert solar energy into chemical energy through solar fuels production.^[^ ^3^ ^]^ In this way, PEC cells offer a renewable alternative to fossil fuels, enhancing energy security and reducing environmental issues. Research in this field concentrates on developing more sustainable and efficient materials to boost their efficiency, stability, and scalability.

A wide range of materials have been developed to be used as photoelectrodes, with particular emphasis on inorganic semiconductors (ISs) such as metal oxides and metal chalcogenides,^[^ ^4^ ^]^ in general cheap and robust. However, despite substantial advances, current energy conversion efficiencies of single materials remain lower than those required for industrial application due to recurring problems such as rapid charge recombination and limited visible light absorption. The most successful strategies to improve the performance of photoelectrodes so far have been: 1) the modification of the optoelectronic properties,^[^ ^5^ ^]^ 2) modifying and functionalizing the surface of the photoelectrode,^[^ ^6^ ^]^ 3) the use of metal cocatalysts,^[^ ^7^ ^]^ 4) the building of heterojunctions formed by two or more ISs,^[^ ^8^ ^]^ and, more recently, 5) the development of hybrid systems composed of inorganic and organic semiconductors.^[^ 9, 10 ^]^ Inorganic–organic heterojunctions (hybrids) are receiving much attention due to their attractive advantages. They combine the high stability and favorable electronic properties of ISs with the tunable optoelectronic properties and flexibility of organic polymer synthesis. Organic polymers offer many opportunities for designing and selecting efficient nontoxic solar energy conversion systems.

Conjugated polymers (CPs) have proven their efficiency in optoelectronic applications such as photovoltaic cells,^[^ ^11^ ^]^ arising as a promising strategy to develop multifunctional layers in photoelectrodes. Their delocalized π‐electron system along the polymer chain facilitates efficient charge transport while providing light absorption capacity, which makes them interesting to improve the properties of some highly researched materials with limitations such as charge recombination or lack of absorption in the visible part of the solar spectrum. Furthermore, the wide variety of functional groups in the building blocks of CPs allows the tuning of their optoelectronic properties in order to optimize charge transport. These advantageous features are achieved without the need for annealing or postpolymerization treatments, allowing for seamless integration of both the absorber and charge transport layer without altering the underlying substrate. Therefore, the formation of a conformal heterojunction with well‐aligned band structures between CPs and inorganic materials offers an effective strategy to reduce charge transport losses in the photoelectrode. In fact, some CPs have already been widely used in PEC cells in hybrid configuration.^[^ 12, 13 ^]^ However, their linear molecular structure with hydrocarbon side chains that makes them easy to process as thin films tends to be unstable in water and under constant lighting due to the reactive αC atom, next to the CP backbone, being vulnerable to radical formation.^[^ ^14^ ^]^ Besides, exocyclic double bonds in the backbone can also be photo‐oxidized in the presence of oxygen‐producing chain scission.^[^ ^15^ ^]^

In particular, conjugated porous polymers (CPPs) have attracted substantial attention, since they maintain the interesting CPs optoelectronic properties while providing intrinsic porosity, larger surface area and improved chemical and structural stability. Besides, their sustained conjugation drives electron delocalization, enabling broad solar spectrum absorption and promoting efficient charge separation and transport.^[^ ^16^ ^]^ However, the utilization of CPPs in PEC cells is not widespread as it is hindered by difficulties in creating high‐quality thin films. The synthesis of CPPs typically yields insoluble powders consisting of polymer particles at the micrometer scale. This poses challenges for preparing stable and high‐quality thin films, affecting light absorption, charge transport, and mechanical stability.^[^ ^16^ ^]^ Consequently, despite some CPPs showing activity toward the photocatalytic hydrogen evolution reaction,^[^ 10, 17, 18 ^]^ oxygen evolution reaction,^[^ ^19^ ^]^ and CO_2_ reduction reaction,^[^ 20, 21 ^]^ their lack of processability restrains their use for PEC and other optoelectronic applications at very‐low‐technology readiness levels.

In this sense, our group is tackling these challenges through bottom‐up nanostructuring synthetic strategies to reduce particle size and dispersity, achieving improved PEC properties.^[^ ^22^ ^]^ These efforts are aimed at enhancing film stability and catalytic properties, which are essential for boosting the efficiency of CPPs in solar energy conversion.^[^ 23, 24 ^]^ In this work, we employ target monomers capable of electrochemically generating a radical cation to successfully synthesize CPP thin films for PEC electrodes through electropolymerization. This simple and cost‐effective method enables precise control over film thickness, ensures homogeneity, and promotes strong covalent bonding between multiple polymer layers and the conductive substrate. Thanks to the fine control of the film deposition layers, the layer thickness can be easily optimized and the transparency of the final film allows the formation of multilayered hybrid systems that maximize light absorption and photoresponse. Moreover, careful choice of monomer units can deliver polymers with photoelectronic properties that enhance intra and intermolecular charge transfer and minimize charge recombination.

In this work, we focus our efforts on the use of two thiophene‐based monomers with potential to be electropolymerized.^[^ ^25^ ^]^ Previous research has demonstrated that thiophene‐based CPPs are effective hole conductors due to their π‐conjugated system and highest occupied molecular orbital (HOMO)–lowest unoccupied molecular orbital (LUMO) alignment relative to other semiconductors which provides a pathway for hole motion with minimal energy required for electron excitation. We have managed to produce high‐quality thin films as part of multilayer CPPs/TiO_2_ hybrid photoelectrodes, proving their synergistic PEC response. Moreover, an exhaustive analysis of the operation of this heterojunction has been carried out through (photo)electrochemical impedance spectroscopy (PEIS) and transient absorption spectroscopy (TAS). These advanced spectroscopies reveal that these hybrid systems enhance both light absorption, by maximizing the part of the solar spectrum usable by TiO_2_, and hole conduction, preventing recombination, reducing charge transfer resistance and increasing the lifetime of photogenerated charges. Overall, the work presented in this manuscript describes the synthesis and preparation of electropolymerizable thiophene‐CPP‐based systems in hybrid photoelectrodes through a simple and cost‐effective method and highlights its promising potential for enhanced solar fuel production through PEC and advanced spectroscopic techniques.

## 2 Results and Discussion

### 2.1 CPP Thin‐Film Synthesis and Characterization

**Figure 1:**
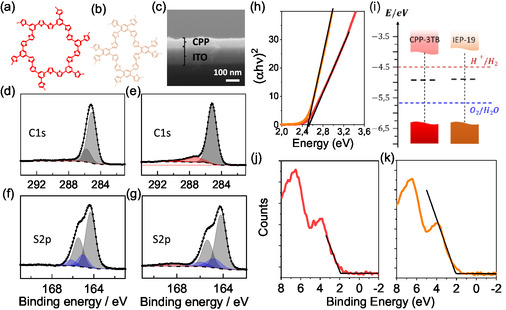
Chemical structure of a) CPP‐3TB and b) IEP‐19. c) FESEM cross‐section of IEP‐19 thin film. d–g) High Resolution XPS spectra of CPP‐3TB (d and f) and IEP‐19 (e and g). h) Tauc plot of CPP‐3TB (red) and IEP‐19 (orange). i) Electronic structure of both CPPs derived from the combination of Tauc plots, XPS and CV. XPS spectra showing the difference between *E*_f_ and VB of j) CPP‐3TB and k) IEP‐19 thin films.

Two thiophene‐based CPPs named CPP‐3TB and IEP‐19 (IMDEA Energy Polymer) were synthesized by electropolymerization using 1,3,5‐tri(2‐thienyl) benzene (3TB) and 1,2,4,5‐tetra(2‐thienyl) benzene (4TB) monomers, respectively (see idealized structures in **Figure** **1**a,b). The detailed synthesis and characterization of the monomers and CPPs can be found in Section S1, S2, Supporting Information. Figure S1, S2, Supporting Information, show the NMR spectra of these monomers that confirm their successful synthesis.

Cyclic voltammetry (CV) was used for electropolymerization (Set‐up scheme in Figure S3, Supporting Information), providing high‐quality, homogeneous, transparent, and insoluble polymeric thin films.^[^ ^25^ ^]^ Figure S4, S5, Supporting Information display the CVs performed to prepare CPP thin films. An inset in each CV shows how the oxidation peak shifts to higher current after each cycle, indicating the continuous growth of the polymeric films (see Figure 1c and Section S3, Supporting Information). The number of cycles, the potential window, and the scan rate of the electropolymerization procedure were optimized by a thickness study (Section S3, Supporting Information). The potentials are chosen according to the oxidation and polymerization potential of the selected monomers.^[^ ^26^ ^]^ The thickness of the polymeric thin films was measured by atomic force microscopy (AFM) (Figure S6, S7, Supporting Information) and field‐emission scanning electron microscopy (FESEM) (Figure 1c). CPP‐3TB and IEP‐19 films thicknesses obtained by this optimized procedure result in 90–110 nm‐thick films. As seen in Figure S6, Supporting Information, increased thickness results in unadhered films. Figure S8, Supporting Information, shows a photograph of a CPP‐3TB polymer sheet where the homogeneity and transparency can be seen.

The chemical composition of the CPP thin films surface was investigated by X‐ray photoelectron spectroscopy (XPS). Figure S9, S10, Supporting Information, show the survey XPS spectra for CPP‐3TB and IEP‐19. At first glance we can confirm the presence of C, S, and O atoms, major elements in polymers. Figure 1d,e shows the high‐resolution XPS (HR‐XPS) spectra in the C1s region of CPP‐3TB and IEP‐19, respectively. Both C1s peaks, centered at 285 eV, have small shoulders at higher binding energies, showing the presence of oxidized C species such as carbonates.^[^ 27, 28, 29 ^]^ S2p HR‐XPS spectra are depicted in Figure 1f (CPP‐3TB) and g (IEP‐19) and both required two sets of p doublets to fit the area under the S2p peak, centered at 165 eV, which is typical of thiophenes.^[^ 27, 28, 29 ^]^ Moreover, IEP‐19 needed an additional pair of peaks to account for a spectral intensity at around 168 eV, which is characteristic of sulfates^[^ 27, 28, 29 ^]^ (Figure S9, S10, Supporting Information). We can see that the proportion of oxygen, albeit small, is larger on IEP‐19 than on CPP‐3TB (9 and 4% respectively), which is in accordance with the presence of more noticeable peaks corresponding to oxidized sulfur and carbon species for IEP‐19 than for CPP‐3TB (see red peaks in Figure 1e,g). The position of the peaks, along with the insoluble nature of the films, confirms the synthesis of CPP‐3TB and IEP‐19 polymers. Morphology and homogeneity were studied by FESEM. The chemical composition was confirmed by the distributed concentration of S and C detected by energy‐dispersive X‐ray spectroscopy (EDS) both in frontal and cross‐section configuration (Figure S11–S14, Supporting Information). The FESEM cross‐section (Figure 1c) confirms a film thickness of around 100 nm, in the same range as that measured by AFM. The frontal SEM images of the polymers exhibited a homogenous distribution of a cauliflower‐like morphology, in which the polymeric 3D network grows in rounded structures. These kinds of aggregates have channels among them that can be beneficial for catalytic applications, as they increase the total surface area, as it has been previously reported.^[^ ^30^ ^]^

The determination of the electronic band diagram is essential for assessing whether these materials possess the thermodynamic capability required to facilitate the intended PEC reactions. In this sense, diffuse reflectance UV–vis–NIR spectroscopy was used to determine the optical bandgap energy by measuring the diffuse reflectance (Figure S15, Supporting Information) and building the Tauc plot represented in Figure 1h (Section S6 in the SI). Two very similar direct transitions of 2.50 ± 0.05 eV were determined. These values confirm that both CPPs are capable of absorbing visible light. The HOMO and LUMO positions were obtained by CV.^[^ ^31^ ^]^ Figure S16, Supporting Information, shows the CVs that reveal a *E*_HOMO_ of 1.5 and *E*_LUMO_ of –1.1 ± 0.1 V_Ag/AgCl_ for CPP‐3TB and *E*_HOMO_ = 1.5 and *E*_LUMO_ = –1.1 ± 0.1 V_Ag/AgCl_ for IEP‐19. The difference between these values gives an electronic bandgap energy of 2.60 ± 0.02 eV, just slightly above the optical ones as it usually happens.^[^ ^32^ ^]^ This difference between the electrochemical bandgap and the optical absorption edge in this kind of polymers can be attributed to the exciton binding energy, which is the energy necessary to bind a photogenerated electron–hole pair (exciton).^[^ 33, 34 ^]^ The small value of around 0.1 eV of the exciton binding energy is characteristic of a large 3D network such as the ones formed in CPP‐3TB and IEP‐19 on the ITO substrate. Finally, the valence band (VB) energy distance to the Fermi level (*E*_F_) was measured by XPS,^[^ ^35^ ^]^ revealing 1.9 and 2.0 ± 0.1 eV for CPP‐3TB and IEP‐19 respectively (Figure 1j,k). In Figure 1i the elucidated electronic band diagram of both polymers is represented along the water‐splitting potentials, revealing the thermodynamic feasibility to carry out these reactions.

### 2.2 Photoelectrochemical Characterization of Polymeric Films

**Figure 2:**
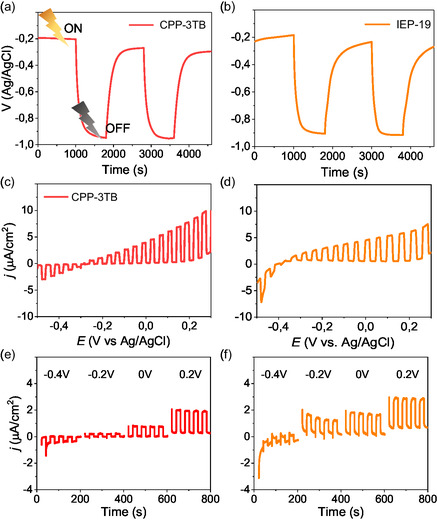
OCP of a) CPP‐3TB and b) IEP‐19 under chopped AM1.5G illumination. LSV and chronoamperometries of c,e) CPP‐3TB and d,f) IEP‐19 under chopped AM1.5G illumination in 0.5 m Na_2_SO_3_. Potential values in (e) and (f) are in V_Ag/AgCl_.

**Figure** **2**a,b display the results of the open‐circuit potential (OCP) measurements under light and dark conditions. When the sample is illuminated, a change of the OCP voltage toward more negative values reveals a n‐type character, which can be related to the big VB‐*E*_F_ distance derived from the analysis of the XPS valence band spectra (Figure 1j–k).

Both polymers exhibit a similar photovoltage: 0.75 V for IEP‐19 versus 0.72 V for CPP‐3TB. The linear sweep voltametry (LSV) performed for both polymers (Figure 2c,d) exhibit positive photocurrents in a wide range of potentials confirming the n‐type character. The acquired photocurrents are in the same order of magnitude, around 7 and 6 μA at 0.2 V_Ag/AgCl_ for CPP‐3TB and IEP‐19 respectively. In addition, chronoamperometries (Figure 2e,f) show similar photocurrents at constant potentials: 1.6 and 2.2 μA at 0.2 V_Ag/AgCl_ for CPP‐3TB and IEP‐19 respectively. The flat band potential, V_FB_,^[^ ^36^ ^]^ can be correlated with the point at which the photocurrents sign switches in the LSV (–0.28 and −0.38 V_Ag/AgCl_ for CPP‐3TB and IEP‐19, respectively). These values, considering a pH of 9 for 0.5 m Na_2_SO_3_ electrolyte, corresponds to *E*_F_ = 0.45 and 0.35 V_RHE_, respectively. The *E*_F_ of both polymers are closer to the conduction band (CB) than to the VB, confirming their n‐type behavior. The difference between VB and *E*_F_ determined by CV and LSV are 1.78 and 1.88 eV for CPP‐3TB and IEP‐19, respectively, very close to the difference calculated by XPS: 1.9 and 2 eV. The small variation between the two methods can be attributed to the small oxidation of the polymers’ surface previously observed in the XPS measurements, which makes it slightly more n‐type than the bulk because of the negative charges provided by the oxygen atoms.

On the other hand, an exploration of the charge transfer across the semiconductor–electrolyte interface was performed by electrochemical impedance spectroscopy (EIS) measurements, which were carried out on the two polymers at 0 V_Ag/AgCl_ in dark and AM1.5G illumination conditions, leading to the Nyquist plots in Figure S17, Supporting Information, which could be fitted by a Randles circuit. Results confirm the capacity of the polymers to act as photoelectrodes, as their charge transfer resistance, *R*_ct_, decreases one order of magnitude upon illumination: from 5000 to 260 kΩ for CPP‐3TB and from 3000 to 370 kΩ for IEP‐19. Moreover, *R*_ct_ is lower for CPP‐3TB, which agrees with its slightly higher measured photocurrents. We can attribute both the lower *R*_ct_, and the consequent higher photocurrents, and slightly higher photovoltage of CPP‐3TB, compared to IEP‐19, to its simpler 3D network.

From the point of view of the theoretical structure of the polymers, it seems that the pores of CPP‐3TB can be larger than those of IEP‐19, allowing the electrolyte to pass more easily into its interior and, therefore, improving the transport of the photogenerated charge to the electrolyte.^[^ ^26^ ^]^

On the other hand, the fact that 3TB has three bonding positions accessible for polymerization while 4TB has four could make it difficult for photoelectrons and holes to reach the back and front of the photoelectrode respectively. Interconnected networks are very useful to improve the stability of polymer films; as seen above, linear structures are very unstable under continuous irradiation conditions, but an overly interconnected structure could hinder the conductivity of the resulting photoelectrodes. We will go into more detail later.

#### 2.2.1 Hybrid CPP‐Based Photoelectrodes

**Figure 3:**
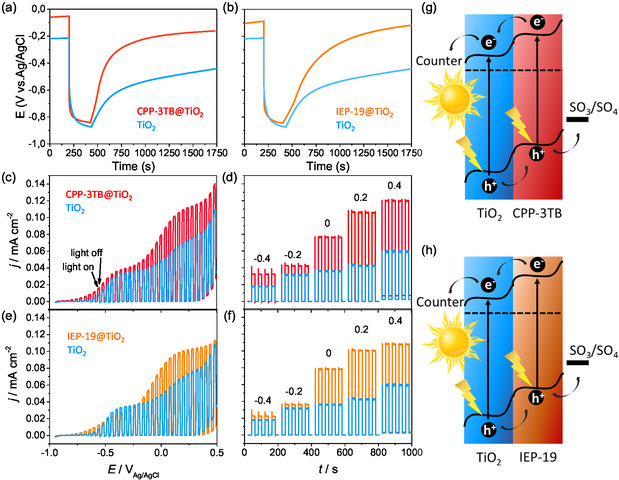
OCP of a) CPP‐3TB@TiO_2_ and b) IEP‐19@TiO_2_ photoanodes compared to bare TiO_2_. c,e) LSV and d,f) chronoamperometries of (c,d) CPP‐3TB@TiO_2_ and (e,f) IEP‐19@TiO_2_ photoanodes compared to bare TiO_2_. All measurements were performed under chopped AM1.5G illumination in 0.5 m Na_2_SO_3_. g,h) Schematic of the charge transfer between polymer‐TiO_2_ taking into account the alignment of the electronic band position of both materials.

As explained before, the combination of semiconductors can provide enhanced PEC performance.^[^ 10, 16 ^]^ TiO_2_ has been one of the most investigated photoanodes due to its high oxidative power, stability, availability and its nontoxicity.^[^ ^37^ ^]^ However, its wide bandgap energy prevents it from absorbing visible light and it also exhibits fast electron–hole recombination and slow charge carrier transfer kinetics.^[^ 38, 39 ^]^ Considering the optoelectronic properties of the synthesized CPPs, it seems that both could act as multifunctional layers in hybrid electrodes with TiO_2_ (see theoretical band alignment and possible charge transfer in **Figure** **3**g,h). These CPP layers will act on the one hand as absorbers of the visible part of the solar spectrum and on the other as photogenerated holes conductors toward the electrolyte, reducing electron–hole recombination.

To demonstrate these hypotheses, two hybrid photoanodes where either CPP‐3TB or IEP‐19 are electropolymerized on top of TiO_2_ were prepared. TiO_2_ thin films were prepared by a sol–gel method based on previous works^[^ 40, 41 ^]^ (more details in Section S8 in the SI). Figure S18, Supporting Information, shows the X‐ray diffraction (XRD) spectra of the prepared TiO_2_ photoelectrode.

Elemental analysis by EDS of the electropolymerized hybrid photoelectrodes and bare TiO_2_ (Figure S20, Supporting Information) shows the homogeneous presence of C, S, Ti, and O. Besides, the morphology of these photoelectrodes shown in the FESEM images consists of the same cauliflower‐shaped agglomerates observed in the samples prepared on ITO. The PEC properties were first analyzed by OCP measurements with chopped AM1.5G illumination (Figure 3a,b), showing the photopotential generated in each photoelectrode. Indeed, both hybrid photoanodes showed higher photopotentials (0.79 ± 0.01 and 0.75 ± 0.01 V for CPP‐3TB@TiO_2_ and IEP‐19@TiO_2_ respectively) than bare TiO_2_ (0.66 ± 0.01 V). LSV measures presented in Figure 3c exhibits an increase in the photocurrent of the hybrid CPP‐3TB@TiO_2_ photoanode, especially toward more positive potentials. Moreover, the chronoamperometries (Figure 3d) show how the photocurrent doubles when CPP‐3TB is electropolymerized on TiO_2_, growing from 0.058 to 0.113 mA cm^−2^ at 0.4 V_Ag/AgCl_. A similar outcome resulted from the study of the IEP‐19@TiO_2_ hybrid photoanode (Figure 3e,f), with the photocurrent almost doubling its value, from 0.058 to 0.105 mA cm^−2^ at 0.4 V_Ag/AgCl_, after the polymer is added.

The calculation of the external quantum efficiency or incident photon‐to‐current efficiency (IPCE) from chronoamperometries can provide insights into the light absorption and charge transfer efficiency of our photoelectrodes at different wavelengths. Figure S21, Supporting Information, shows the IPCE at 0.4 V_Ag/AgCl_ of both hybrids and bare TiO_2_. The maximum of IPCE for bare TiO_2_ is centered around 320 nm, which corresponds to 3.9 eV, higher than the bandgap energy of anatase (≈3.2 eV). This difference can account for the overpotential that is required to drive the reaction as fast as possible.^[^ ^42^ ^]^ In the case of both hybrids, the maximum IPCE grows and shifts toward visible light with respect to TiO_2_ alone. This change can be attributed to the smaller bandgap energy of the electropolymerized CPPs compared with TiO_2_ and to an improved charge transfer. The integration of IPCE values at the wavelengths under study is higher for CPP‐3TB@TiO_2_ (9.4%) and IEP‐19@TiO_2_ (7.5%), than for TiO_2_ (7.2%), showing how the presence of the CPP layer makes a difference in the efficiency. The photocurrents exhibited by the CPP‐3TB and IEP‐19 electrodes alone are an order of magnitude lower than the photocurrent enhancement in the hybrid photoanodes with respect to TiO_2_. This means that the enhancement does not occur only due to the sum of their photocurrents: a synergistic effect is taking place between the organic and inorganic thin films in the hybrid photoanodes, as idealized in Figure 3g,h, demonstrating that these electronic structures have facilitated the enhancement in charge transfer and light absorption.

### 2.3 Photophysics Mechanism Insights

**Figure 4:**
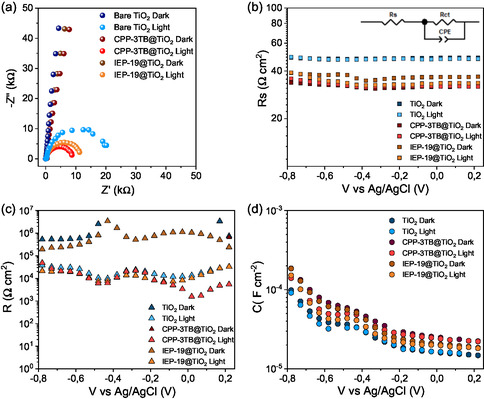
PEIS results. a) Nyquist plots at 0 V versus Ag/AgCl. Extracted parameters from the fitting of the PEIS raw data along the whole applied potential window: b) series resistance (*R*_s_), c) charge transfer resistance (*R*_ct_), and d) capacitance.

To improve our understanding of the physical processes behind the performance of the prepared hybrid photoanodes, a combination of PEIS^[^ 43, 44, 45 ^]^ and TAS has been performed herein.^[^ 46, 47 ^]^ **Figure** **4**a shows the measured Nyquist plots in the samples under study under both dark and 1 Sun illumination conditions at 0 V versus Ag/AgCl. Notice that in all cases the size of the arcs decreased upon illumination, as expected in a photoelectrode.^[^ 48, 49 ^]^ In the case of TiO_2_ alone, a larger semicircle is observed than in the case of both hybrids, which, as will be detailed later, may be associated with a higher resistance to the charge transfer. Since a single arc was observed in the Nyquist plots, a simple Randles’ circuit (inset, Figure 4b) was employed to fit the raw data.^[^ ^50^ ^]^ This equivalent circuit contains three elements: a series resistance (*R*_s_), as a consequence of the wiring and the ITO substrate, a charge transfer resistance (*R*_ct_), and a capacitance (C) associated with the photoanode/electrolyte interface. After fitting the PEIS data along the whole potential window with the explained equivalent circuit, different parameters can be extracted. Figure 4b shows the values of the *R*_s_, showing a flat behavior with the potential, with values between 30 and 40 Ω cm,^[^ ^2^ ^]^ along the whole window.^[^ 51, 52 ^]^ Notice that the hybrid samples showed slightly lower values of the *R*_s_ than the bare TiO_2_ photoanode along the whole window. On the other hand, the extracted *R*_ct_ values are shown in Figure 4c. First, in all the cases a clear decrease in the *R*_ct_ upon illumination is shown. Also, the hybrid samples showed lower values of the *R*_ct_ under both dark and 1 Sun illumination, in good agreement with the higher observed photocurrents shown in Figure 3. Additionally, the CPP‐3TB sample shows a lower *R*_ct_ than the IEP‐19, and both show lower values than bare TiO_2_, indicating enhanced charge extraction of the photogenerated holes to the electrolyte to carry out the oxidation.^[^ ^7^ ^]^

On the other hand, Figure 4d shows the extracted capacitances in the analyzed samples. As it can be observed, all the samples show a peak around −0.5 V versus Ag/AgCl. This peak can be associated with the Ti^3+^/Ti^4+^ redox couple,^[^ ^53^ ^]^ due to presence of Ti^3+^ interstitials in the TiO_2_ layer.^[^ ^54^ ^]^ Additionally, a decrease in the capacitance is observed in all the samples with the applied potential as a consequence of an enhanced charge extraction at this region.^[^ 55, 56 ^]^ It is also interesting that the CPP‐3TB shows a higher capacitance than the IEP‐19, and both show higher values of the capacitance than bare TiO_2_. This phenomenon can be attributed to a higher density of photogenerated holes in the hybrid photoanodes compared with bare TiO_2_, as it has been previously reported in other photoelectrocatalytic systems.^[^ ^6^ ^]^

**Figure 5:**
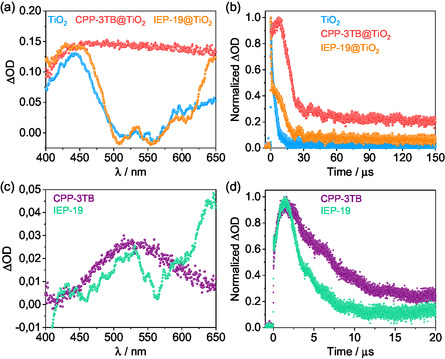
a) TAS ( λ_exc_ = 355 nm, 300 μJ) and b) decay traces (λ_exc_ = 355 nm, λ_mon_ = 460 nm, 300 μJ) for TiO_2_ (blue), CPP‐3TB@TiO_2_ (red), and IEP‐19@TiO_2_ (orange) in deaerated water. ΔOD is the optical density. c) TAS (λ_exc_ = 355 nm, 300 μJ) and d) decay traces (λ_exc_ = 355 nm, λ_mon_ = 530 nm, 300 μJ) for CPP‐3TB (purple) and IEP‐19 (green) polymers. All samples were measured as thin films in deaerated water.

In order to shed light on the electron (e^−^)–hole (h^+^) pair recombination rate and its influence on the photoelectrode performance, TAS measurements were performed. First, transient absorption (TA) spectrum of TiO_2_ exhibited a main band at around 460 nm with a shoulder at 650 nm, which corresponds to the hole and electron signals, respectively (**Figure** **5**a, blue trace).^[^ 39, 57, 58, 59, 60 ^]^ Second, both CPP‐3TB and IEP‐19 polymers exhibited a broad TA with a main band at around 530 nm or 530/650 nm for CPP‐3TB and IEP‐19 respectively (Figure 5c). It has been reported that the triple–triplet absorption for phenylene‐bridged oligothiophene range from 450 to 620 nm depending on the substituent position.^[^ 61, 62 ^]^ Compared to bare TiO_2_, CPP‐3TB@TiO_2_ showed a broad band covering all the spectral window (Figure 5a, red trace). A noticeable increase in the transient absorption is indicative of a slower e^−^–h^+^ recombination, improving the charge transport ability within the hybrid photoanode. This is in agreement with the results obtained from the PEIS results and with our previous works in particulate photocatalytic systems composed of TiO_2_ and CPPs.^[^ ^63^ ^]^ On the other hand, IEP‐19@TiO_2_ (Figure 5a, orange trace) exhibited a moderate increase in the transient absorption at 460 and 650 nm bands. This result suggests that the charge transfer is more moderate than in CPP‐3TB. Furthermore, the transient absorption maximum for both hybrids was centered at around 450 nm. This fact could be attributed to two aspects: 1) a large contribution from the inorganic counterpart in the heterojunction is present, which is enhanced from the charge transfer, and 2) based on the literature, oxidized species from thiophene derivatives (mainly radical cation) generates TA bands centered at 450 nm.^[^ 64, 65 ^]^

Then, analysis of transient lifetimes (*τ*) in the range of ns‐to‐ms timescale at λ_mon_ = 460 nm for TiO_2_ and its corresponding hybrids was performed (Figure 5b). TiO_2_ was fitted with a monoexponential function exhibiting a lived lifetime of 1.3 μs (Figure S22a, Supporting Information), while CPP‐3TB@TiO_2_ experienced a huge increase of τ in three consecutive steps (Figure S22b, Supporting Information): two contributions of 8 and 165 μs, respectively, and third component above 3 ms. Compared to the bare TiO_2_, this long‐lived transient demonstrates much slower electron–hole recombination of the photogenerated carriers, which results in a better candidate as a photoanode. The hybrid IEP‐19@TiO_2_ also showed a huge increase in τ, obtaining values of 386 ns, 11.3 μs and 1.3 ms, respectively (Figure S22c, Supporting Information). These all results probe that hybrids exhibit a slower electron–hole pair recombination, improving their PEC performance, it being higher for CPP‐3TB@TiO_2_. For pristine polymers, CPP‐3TB exhibited longer lifetime than IEP‐19 (5.8 and 2.6 μs, respectively, Figure 5d), which also contributes to better charge transfer at the heterojunction interface.

## 3 Conclusion

Two homogeneous CPP thin films, CPP‐3TB and IEP‐19, have been successfully synthesized via electropolymerization. An exhaustive PEC analysis revealed that CPP‐3TB and IEP‐19 are photoactive and exhibit very similar optoelectronic properties, with CPP‐3TB showing slightly higher photoresponses and lower *R*_ct_. Hybrid thin films were synthesized and characterized using TiO_2_ as the IS material, as their characterized electronic structure should facilitate the charge transfer across the hybrid interfaces. Their PEC characterization reveals a synergistic effect, that includes increased photovoltages, photocurrents, and IPCE.

PEIS and TAS studies elucidated the superior performance of these CPPs@TiO_2_ systems. EIS analysis revealed a marked reduction in *R*_ct_ under illumination, demonstrating improved charge extraction capabilities for both hybrid photoanodes. On the other hand, TAS indicated a slower recombination rate of photogenerated electron–hole pairs, thereby proving the enhanced charge carrier dynamics within both hybrid photoelectrodes.

Comparing the two studied polymers, the lower *R*_ct_ and the higher C for CPP‐3TB highlight its superior charge transfer and photogeneration of carriers, respectively, which are crucial for efficient solar energy conversion. On the other hand, the longer transient lifetimes observed by TAS in CPP‐3TB@TiO_2_ indicate a slower recombination rate in this system in comparison to IEP‐19@TiO_2_. Since the optoelectronic properties of both CPPs are very similar, the observed differences can be attributed to the different structure of these CPPs, which can have a significant effect on the conductivity of photogenerated charges. The TB3 monomer, with its highly symmetric design, forms a more ordered polymer structure, promoting an effective overlap of π orbitals along the conjugated backbone. This alignment could enhance charge delocalization and facilitate a smoother motion of photogenerated electrons and holes, resulting in higher charge mobility and better conductivity. In contrast, the lower symmetry of TB4 structure may tend to produce a more disordered polymer, which disrupts π orbital overlap and creates energy barriers that hinder charge transport. This resulting structure–performance relationship provides key guidance for future synthetic strategies.

This study showcases the vast potential of the electropolymerization method for constructing PEC organic materials for solar energy conversion, offering a promising avenue for synthesizing a wide array of conductive, light‐absorbing polymers, marking the beginning of its exploration in the PEC field. The synthetic flexibility could be used to synthesize both anode and cathode photoelectrodes, while the employment of stabilization strategies, such as conformal thin films or passivation layers, could enhance even further the performance of the resulting hybrid organic–inorganic photoelectrodes and result in high‐performance tandem PEC cells for future solar energy conversion applications.

## Conflict of Interest

The authors declare no conflict of interest.

## Author Contributions

**Elena Alfonso‐González**: data curation (equal); formal analysis (supporting); investigation (lead); writing—original draft (equal); writing—review editing (supporting). **Miguel Gomez‐Mendoza**: formal analysis (supporting); investigation (supporting); writing—original draft (supporting). **Carmen G. López‐Calixto**: investigation (supporting). **Miguel García‐Tecedor**: data curation (supporting); investigation (supporting); writing—original draft (supporting). **Ignacio J. Villar‐García**: data curation (supporting); investigation (supporting); writing—original draft (supporting); writing—review editing (supporting). **Freddy Oropeza**: data curation (supporting); investigation (supporting). **Marta Liras**: formal analysis (supporting); methodology (supporting); supervision (supporting); writing—original draft (supporting); writing—review editing (supporting). **Mariam Barawi Moran**: conceptualization (lead); data curation (equal); funding acquisition (equal); investigation (supporting); methodology (equal); supervision (lead); writing—original draft (equal); writing—review editing (lead). **Víctor A. de la peña O´Shea**: funding acquisition (equal); supervision (supporting); validation (supporting); writing—review editing (supporting).

## Supporting information

Supplementary Material

## Data Availability

The data that support the findings of this study are available from the corresponding author upon reasonable request.
