# Supplementary material for: Hybrid Photoelectrodes Based on Electropolymerized Conjugated Porous Polymers for Enhanced Solar Energy Conversion
Source: Small Sci. 2025 Feb 20;5(6):2400623. doi: 10.1002/smsc.202400623 (PMC12168630; doi:10.1002/smsc.202400623)
Supplement: Supplementary file 1 — Supplementary Material [file SMSC-5-2400623-s001.pdf]

Supporting Information

Hybrid Photoelectrodes Based on Electropolymerized Conjugated Porous Polymers for Enhanced Solar Energy Conversion

Elena Alfonso-González[a,b], Miguel Gomez-Mendoza[a], Carmen G. López-Calixto[a,c], Miguel García-Tecedor[a], Ignacio J. Villar-García[d], Freddy Oropeza[a], Marta Liras[a], Mariam Barawi*[a] and Víctor A. de la Peña O´Shea*[a]

[a] Photoactivated Processes Unit, IMDEA Energy Institute, Avda. Ramón de la Sagra, 3. 28935 Móstoles (Madrid) Spain

[b] Current Address: Institute of Ceramics and Glass, CSIC, Campus de Cantoblanco, 28049 Madrid, Spain

[c] Current Address: Peace Research Institute Frankfurt (PRIF), Department of International Security, Baseler Strasse 27-31, 60329, Frankfurt am Main, Germany.

[d] San Pablo CEU University, Faculty of Pharmacy, Department of Chemistry, Montepríncipe Urbanization 28668 Boadilla del Monte (Madrid), Spain.

Keywords: Photoelectrochemistry • Conjugated porous polymers • hybrid photoelectrodes • Solar energy conversion • Electrochemical Impedance Spectroscopy • Transient Absorption Spectroscopy

S1. Synthesis and characterisation of CPP-TB3 and IEP-19 monomers

<u>Synthesis of Monomers</u>

The first step to prepare a polymeric thin film is the design and synthesis of small molecules used as building blocks or monomers. Thus, 3TB and 4TB were synthesized by means of Stille cross coupling reaction. Stille Coupling is an organic C-C bond forming reaction which is very useful to synthesize a wide range of products.[1] The starting compounds must be a stannane (nucleophile, $\mathrm{RSnR''_3}$) and a halide or pseudohalide (electrophile, $\mathrm{R'-X}$). Convenient X-substituents can be Cl, Br, I or OTf (triflate, $R{-}OSO_2CF_3$). Moreover, there are few limitations for the R-groups, with Ar, vinyl, alkenyl, alkynyl or phenyl as some examples. Also, a palladium catalyst is needed. Thus, the reaction occurs as follows:

$$\mathrm{R'-X+RSnR''_3}\xrightarrow{\mathrm{Pd-Cat}}\mathrm{R'-R+XSnR''_3} \qquad \textit{equation } S1$$

<u>Synthesis of 1,3,5-tri(2-thienyl)-benzene (3TB)</u>

The monomer 3TB was synthesized by the Stille Coupling following the method first reported by Brusso *et al*.[2] (see Scheme S1.). 1,3,5-tribromobenzene (3.00 g, 9.53 mmol), 2-(tributylstannyl) thiophene (12.1 mL, 38.12 mmol) and $Pd(PPh_3)_4$ (440 mg, 0.38 mmol) were placed in a Schlenk tube in DMF (10 mL). The mixture was stirred at 130 ºC under an inert atmosphere during 42 hours. A white precipitate was formed.

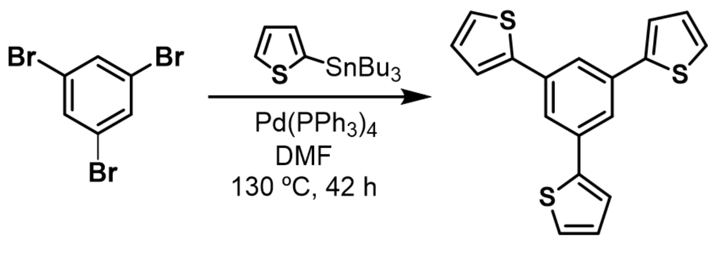

3TB

Scheme S1. *Synthesis of 3TB.*

The final product was purified by recrystallization in ethyl acetate and collected by filtration. 2.29 g of the monomer 3TB were obtained with a yield of 74 %. ^{1}H-NMR (400

MHz, d-Chloroform) δ 7.74 (s, 3H), 7.42 (d, *J*=3.6 Hz, m, 3H), 7.34 (d, *J*=5.1 Hz, 3H), 7.13 (dd, *J*=5.1 and 3.7 Hz, 3H) (Figure S1).

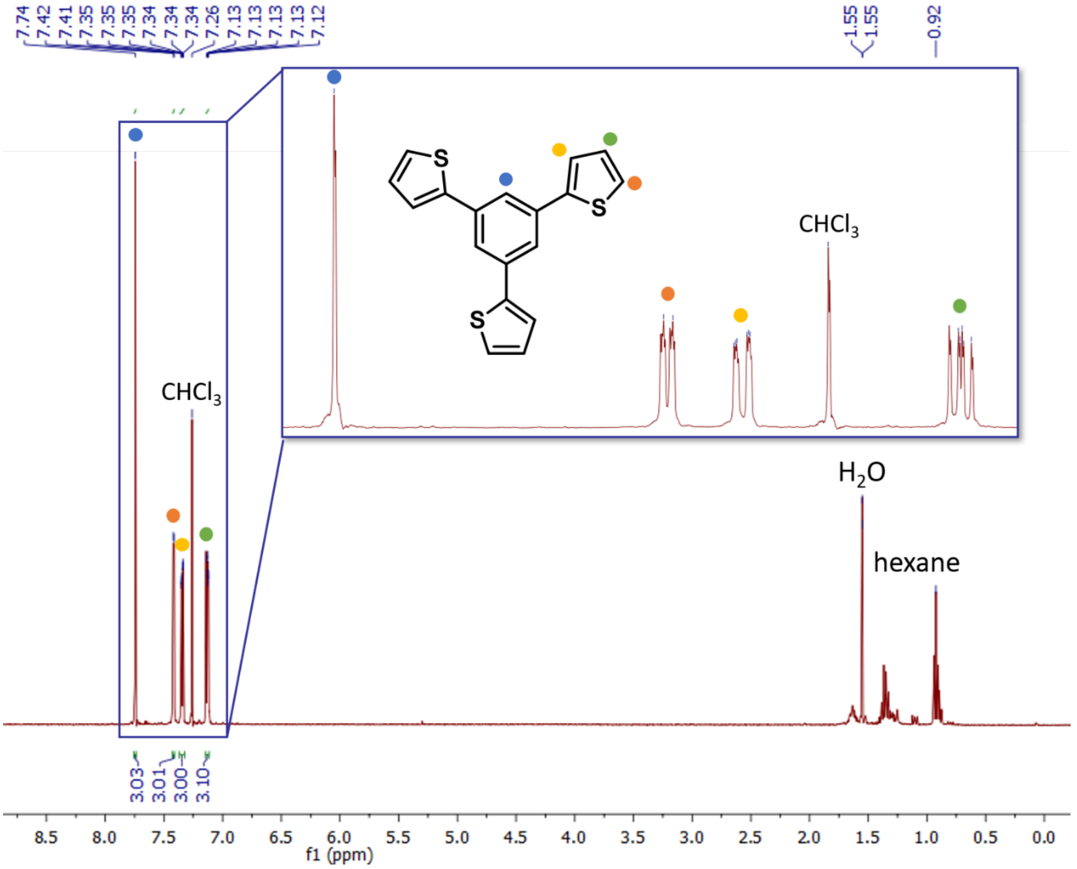

Figure S1. *1H-NMR spectra of 3TB.*

Synthesis of 1,2,4,5-tetra(2-thienyl)-benzene (4TB).

The monomer 4TB was synthesized following the same method reported by Brusso *et al*.[2] (see Scheme S2.). First, 1,2,4,5-tetrabromobenzene (2.30 g, 5.84 mmol), 2-(tributylstannyl) thiophene (9.3 mL, 29.20 mmol) and $Pd(PPh_3)_4$ (266 mg, 0.23 mmol) were placed in a Schlenk tube in DMF (5 mL). The mixture was stirred at 130 ºC during 18 hours under an inert atmosphere. A white precipitate was formed. It was purified by recrystallization in ethyl acetate and collected by filtration. The monomer 4TB (2.17 g) was obtained with an excellent yield (92 %). ^{1}H-NMR (400 MHz, d-Chloroform) δ 7.66 (s, 2H), 7.30 (dd, 4H), 6.97 (m, 8H) (Figure S2).

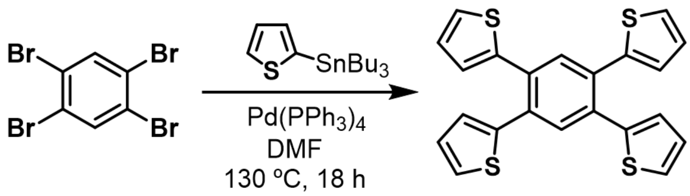

4TB

Scheme S2. *Synthesis of 4TB.*

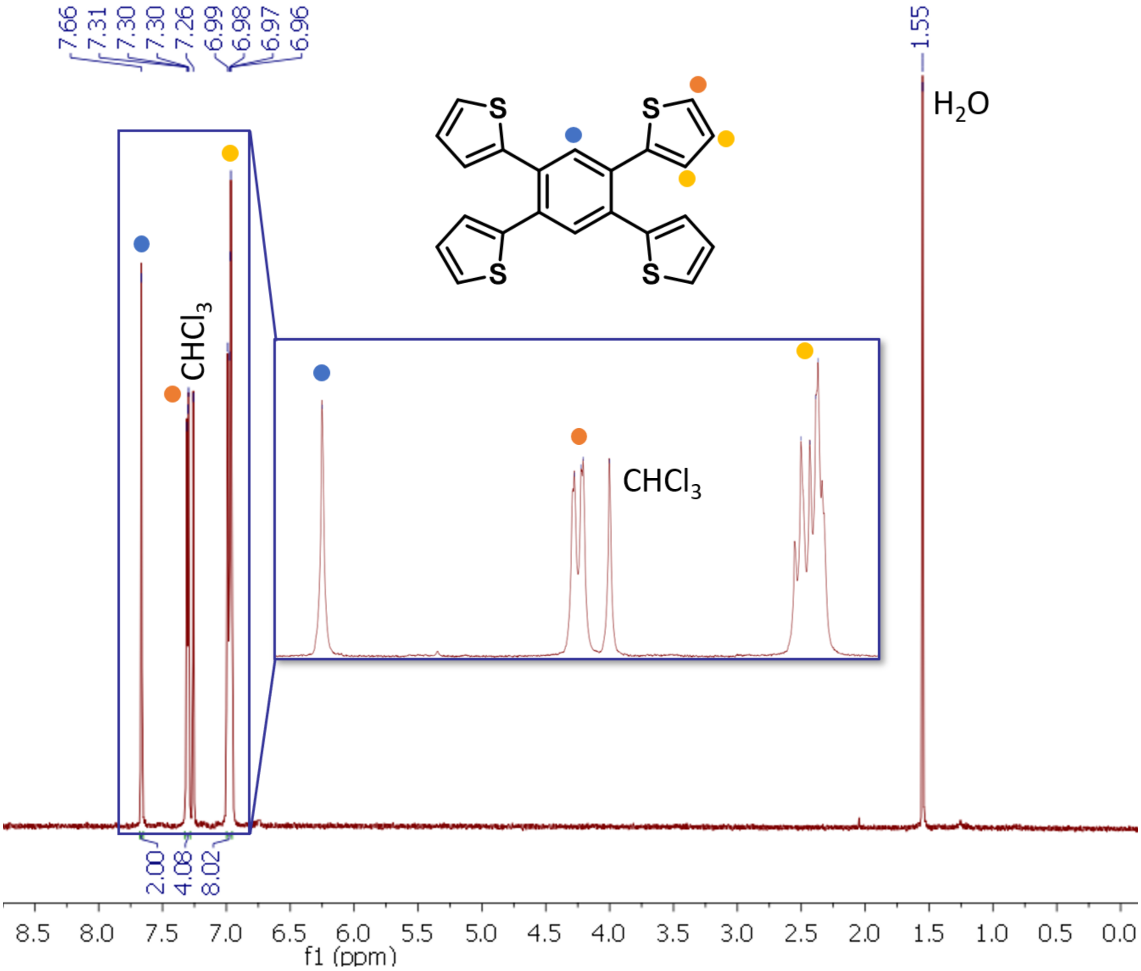

Figure S2. *^{1}H-NMR spectra of 4TB.*

<u>Nuclear magnetic resonance (NMR)</u>

NMR spectra were recorded with a Bruker Model AV300 spectrometer (Larmor frequencies of 300 MHz for ^{1}H) for liquids using $CHCl_3$ as internal reference (7.26 ppm).

Fourier Transform Infrared (FTIR) Spectroscopy

FTIR spectra were recorded on a Thermo-Scientific model Nicolet 6700 spectrometer and are reported in terms of the frequency of absorption (cm^{-1}). The success in the cross-coupling reaction was confirmed with the detection of C-C bonds by FTIR. The polymer spectrum shows the characteristic C-C stretching band located at 2183 cm^{-1} as well as C-S band located at 1080 cm^{-1} while the C-CH stretching band from the starting material ($n_{C\text{-}CH}$ =3280 cm^{-1}) has disappear. Also, the 1,3,5 aromatic substitution of the polymer due to the structural moiety was confirmed by the pattern of the peak bellow 1000 cm^{-1}.

S2. Synthesis of Polymeric Thin Films by Electropolymerisation

The monomers must react among each other and get anchored to a substrate to form a polymeric thin film. In this work, this was achieved by electropolymerisation. Electropolymerisation allows to synthesise a polymer directly on a conductive substrate, obtaining homogenous thin polymeric films.[3,4] It can only be used with monomers that can give a radical ion, such as carbazole, pyrrole or thiophene, which will form covalent bonds among the monomer radicals and between them and the substrate when a certain potential is applied.[5–7] If the monomer has more than two anchoring points, as happens with 3TB and 4TB, the polymer could be a Conjugated Porous Polymer (CPP) with a 3D structure. The typical setup involves the use of an electrochemical cell with three electrodes (Figure S3), a working electrode (WE), a counter electrode (CE), and a reference electrode (RE), which are immersed in an electrolyte solution that contains the monomer. The WE is the one where the polymer will form, with the current flowing between the WE and the CE while the applied potential is measured between the WE and the CE.

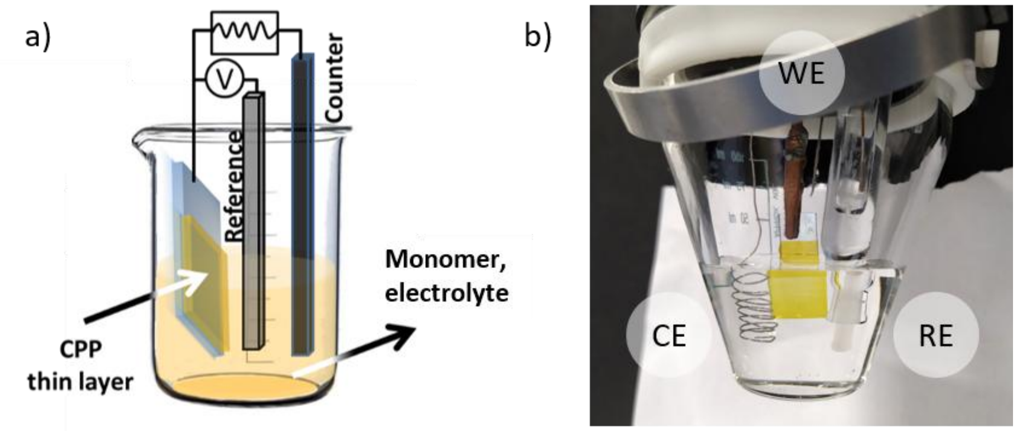

Figure S3. *a) Scheme and b) picture of the electropolymerisation setup.*

There are two possibilities, either to apply the potential at which the radical formation occurs and therefore electropolymerisation takes place for a certain time or to do a cyclic voltammetry (CV) up to that potential for several cycles until the desired polymer thickness is obtained.[4,7,8] In this work, the second procedure was followed, stablishing a relationship between the number of cycles and the thickness of the polymeric films.

Synthesis of CPP-3TB (conjugated porous polymer 3,5-tri(2-thienyl)-benzene) and IEP-19 (IMDEA Energy Polymer 19, conjugated porous polymer 1,2,4,5-tetra(2-thienyl)-benzene).

All photoelectrodes were prepared on indium tin oxide (ITO) coated glass, which was first cleaned in a 1:1 acetone and water mixture for 10 min in an ultrasonic water bath and then dried with a low lint wipe. Then, the ITO coated glass substrate was masked with Kapton tape so that only a 1 cm^2 surface is in contact with the electrolyte solution.

For the electropolymerisation of the monomers, 3TB and 4TB, to obtain CPP-3TB and IEP-19 respectively, a modified method based on the work reported by Gu *et al.*[8] , who synthesized CPP-3TB before us, was used (see schemes S3 and S4). On the contrary, IEP-19 is an original polymer and was synthesized here for the first time. Solutions of 3 mM of monomer and 0.1 M nBu_4NClO_4 as an electrolyte in propylene carbonate were used. The procedure in both cases was as follows. A conical glass cell containing 25 mL of the corresponding solution was purged with Ar for 10 min (see Figure S3b). The working electrode (WE) was the masked ITO substrate where the electropolymerisation takes place, the reference electrode (RE) was Ag/AgCl (3 M KCl) and the counter electrode (CE) was a Pt wire. Once the cell is purged, a CV from 0 to 1.5 $V_{Ag/AgCl}$ at a rate of 0.05 V/s and 14 cycles was performed in order to obtain a homogeneous film of the polymer. The same electropolymerisation method was used to prepare hybrid photoelectrodes.

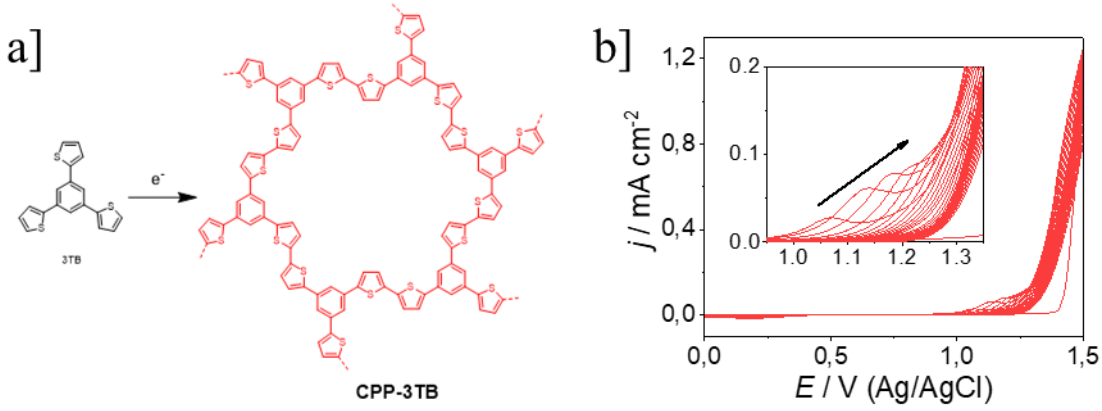

Scheme S4. a) Scheme of the synthesis of CPP-3TB. b) Cyclic voltammetry of CPP-3TB electropolymerisation.

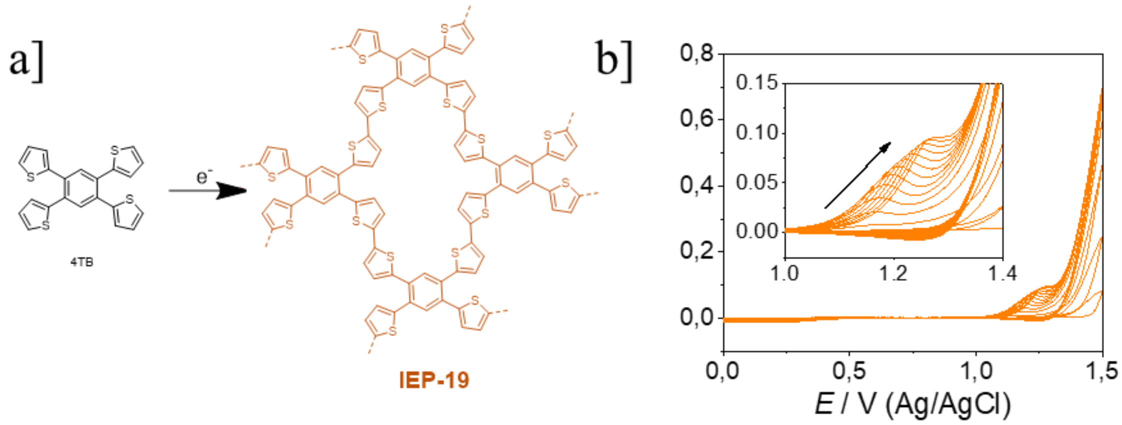

Scheme S5 *a) Scheme of the synthesis of IEP-19. b) Cyclic voltammetry of IEP-19 electropolymerisation.*

S3. Conjugated Porous Polymer (CPP) thin film optimisation

The thickness of the polymeric thin films prepared with different number of CV cycles and potential ranges were measured by AFM (Figures S4 and S5) and FESEM. This way, a correlation between the number of cycles and the thickness of the film could be made. CPP-3TB and IEP-19 films thicknesses varied from 40 to 250 nm depending on the potential range and number of cycles performed (Figures S6c and S7c). The lowest potential applied was always 0 $V_{Ag/AgCl}$ for every CV, while the top potential varied from 1.5 to 1.7 $V_{Ag/AgCl}$. These potentials are chosen according to the oxidation and polymerisation potential of the selected monomers.[8] In Figure S4c we can see how the film thickness increased faster with the number of cycles for electropolymerisation performed at a higher maximum potential. This happens because increasing the applied potential increases the current density generated exponentially, and, therefore, the number of monomer radicals prepared to be electropolymerised. In this sense, following this strategy may seem more efficient. However, this possibility was analysed and it was found that CPP-3TB films with no more than 6 cycles could be achieved with a potential range of 0-1.7 $V_{Ag/AgCl}$, as they would collapse and detach due to their high thickness (Figure S6d and e). The rapid growth at this potential produces thicknesses that are too large to result in stable films as observed in Figure S6d and S6e. For this reason, a 0-1.5 $V_{Ag/AgCl}$ range was chosen as the standard procedure, as more stable and a finer control of the thin film thickness can be achieved this way. Moreover, a study of the relationship of thicknesses with the photopotential was carried out in order to maximize the latter, and we could see higher photopotentials with increasing cycles

for both polymers, with the 0 to 1.5 $V_{Ag/AgCl}$ potential range as the one that gave the samples with the largest photopotentials.[9] For these reasons, we chose 14 cycles from 0 to 1.5 $V_{Ag/AgCl}$ as the standard electropolymerisation procedure to prepare our CPPs samples during this work.

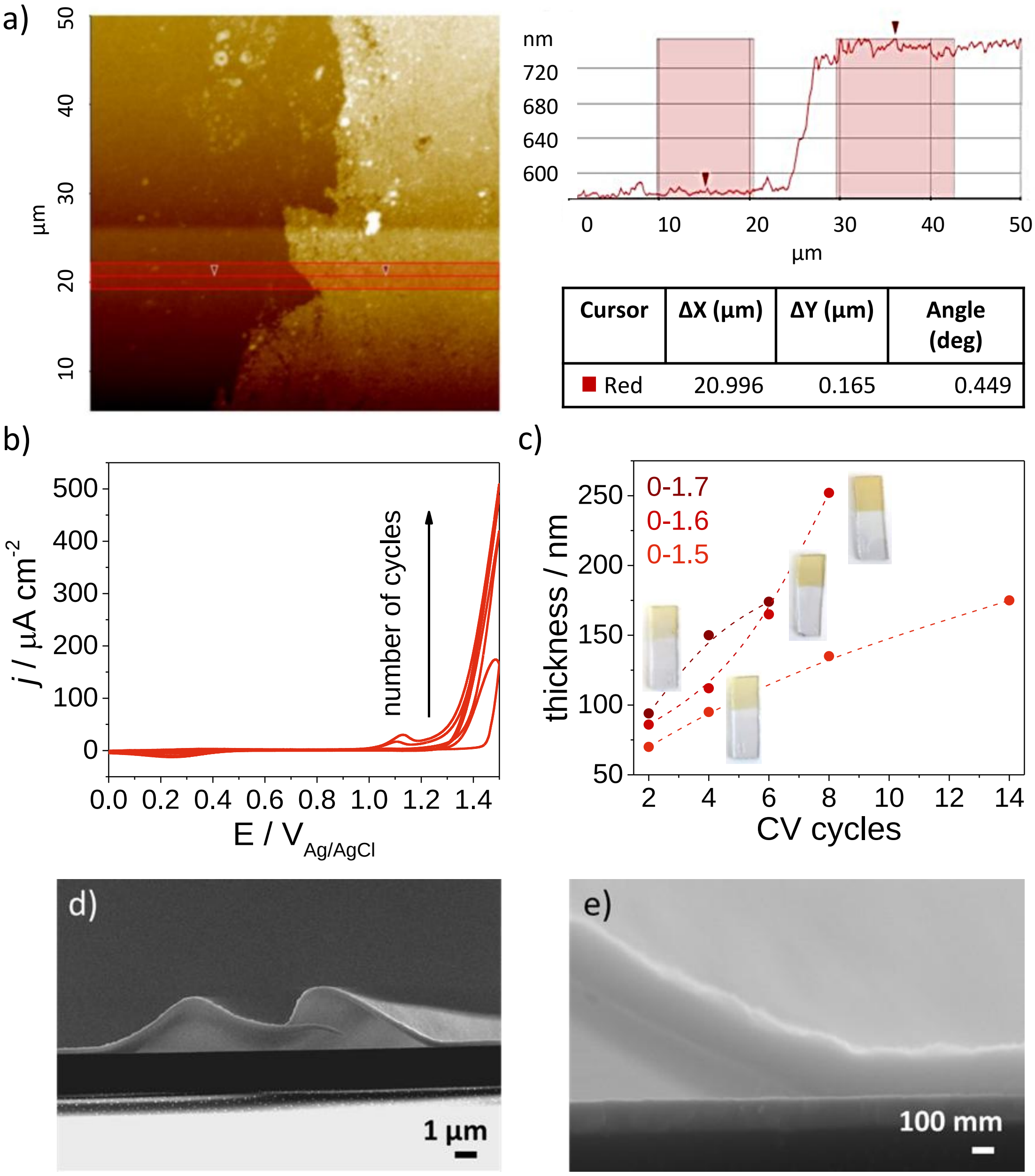

Figure S6. a) Example of an AFM image and thickness measurement of a CPP-3TB thin film. b) 4-cycle CV from 0 to 1.5 VAg/AgCl corresponding to the electropolymerization of 3TB to synthesize a CPP-3TB thin film. c) Thickness study of CPP-3TB samples with different number of CV cycles and potential ranges (in $V_{Ag/AgCl}$),

with pictures of the thin films obtained for the 0-1.6 $V_{Ag/AgCl}$ range. d) and e) cross sections of CPP-3TB thin films obtained for the 0-1.7 $V_{Ag/AgCl}$ range.

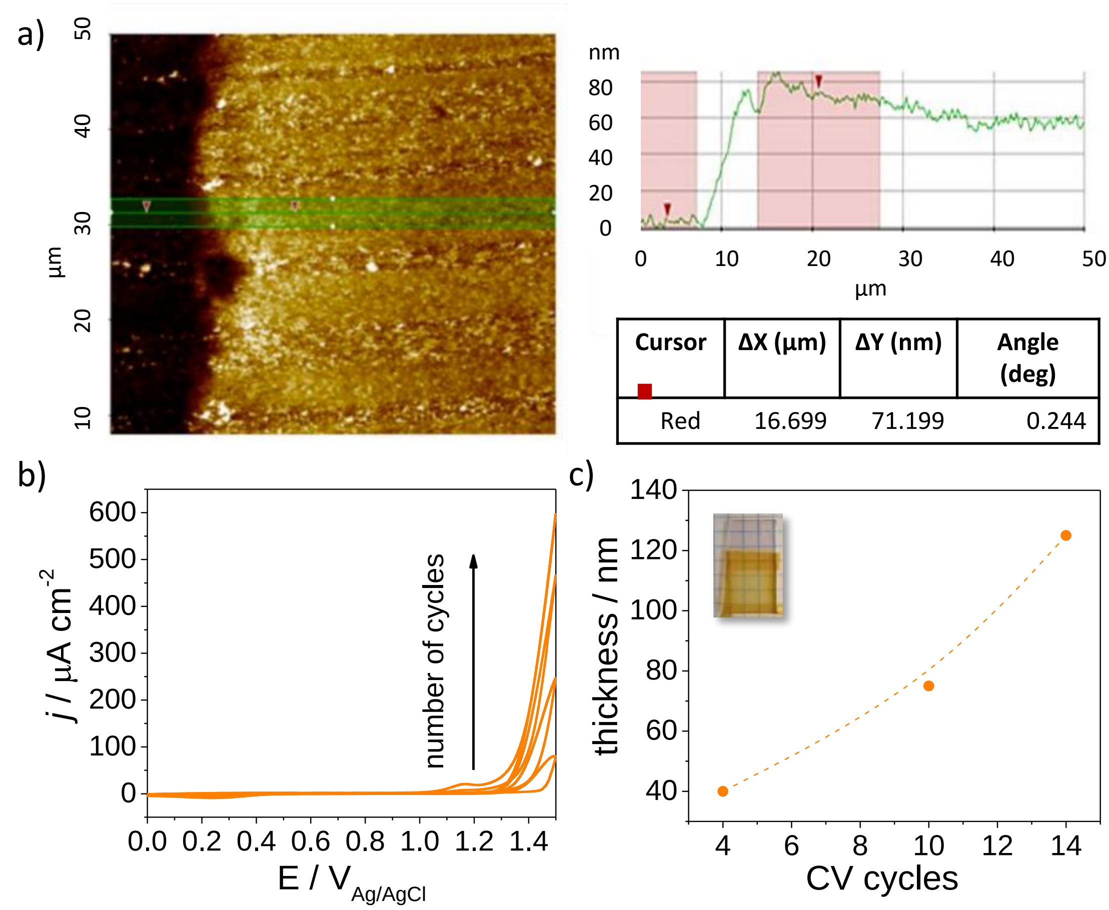

Figure S7. a) Example of an AFM image and thickness measurement of an IEP-19 thin film. b) 4-cycle CV from 0 to 1.5 $V_{Ag/AgCl}$ corresponding to the electropolymerization of 4TB to synthesize an IEP-19 thin film. c) Thickness study of IEP-19 samples with different number of CV cycles from 0 to 1.5 $V_{Ag/AgCl}$ with a picture of a 14-cycle IEP-19 thin films with a Kapton tape mask.

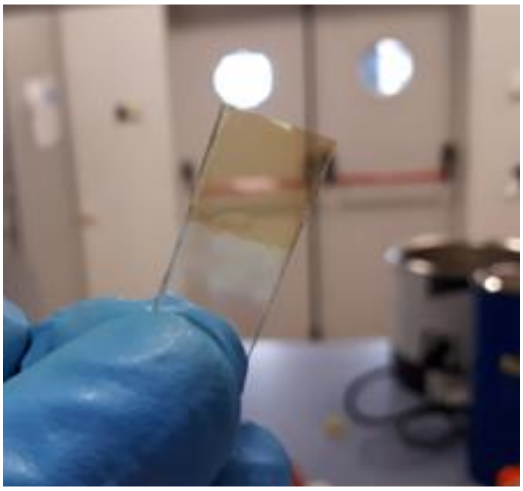

Figure S8. Image of the electropolymerised CPP-3TB thin film.

S4. X-Ray Photoelectron Spectroscopy (XPS) survey analysis

The position of the peaks in the survey scan, along with the insoluble nature of the films, confirm the synthesis of CPP-3TB and IEP-19 polymers.

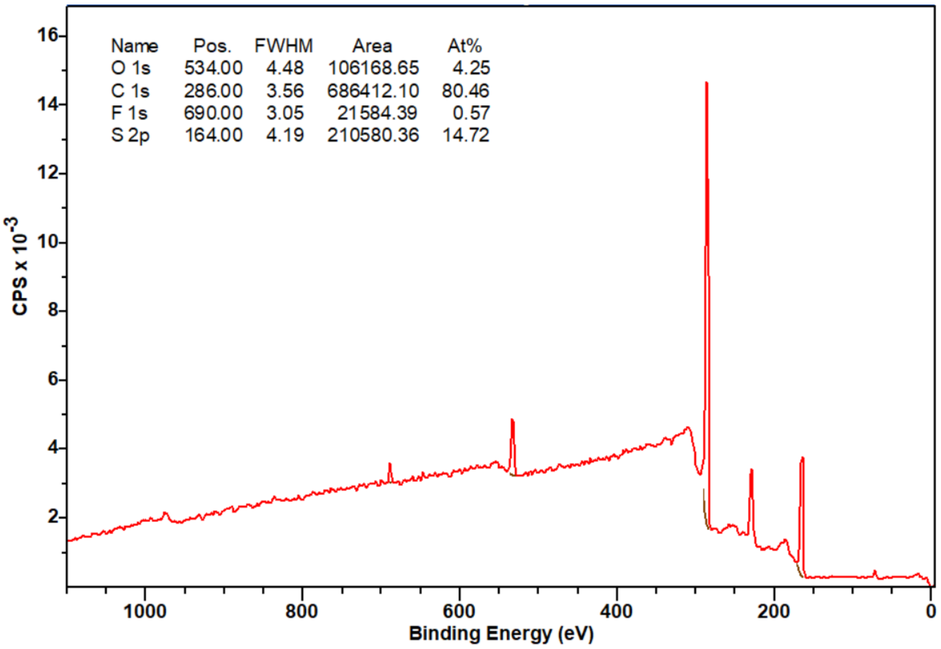

Figure S9. *XPS survey spectrum and chemical analysis of a CPP-3TB thin film.*

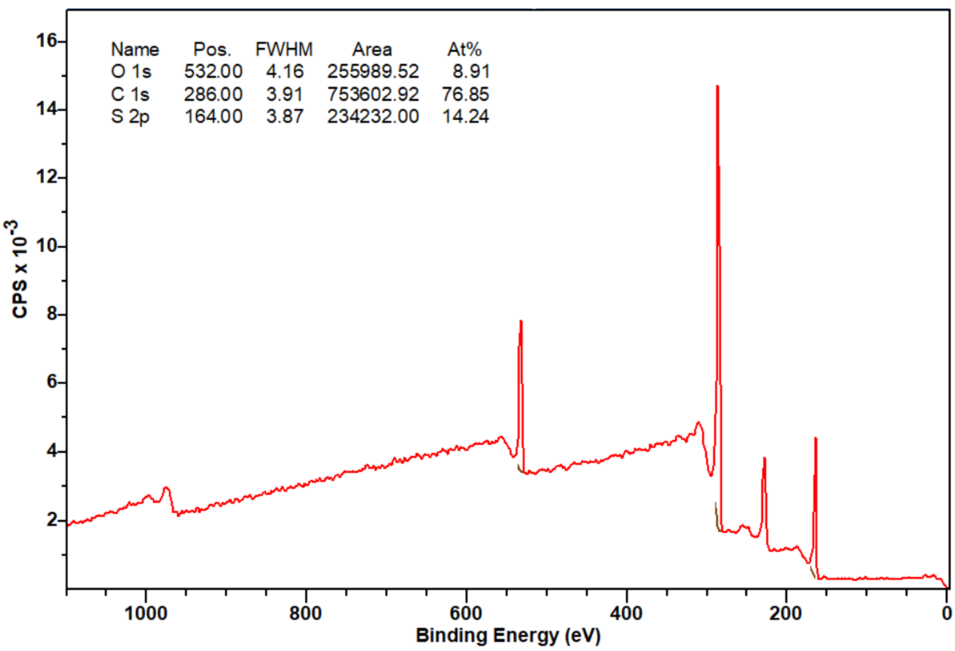

Figure S10. *XPS survey spectrum and chemical analysis of an IEP-19 thin film.*

S5. FESEM for morphological analysis

<u>Field Scanning Electron Microscopy (FESEM) analysis of CPP thin films</u>

Morphology, homogeneity and thickness of the electropolymerised films was studied by Field Emission Scanning Electron Microscopy (FESEM). The chemical composition of the polymer was confirmed by the distributed concentration of S and C detected by Energy Dispersive X-ray Spectroscopy (EDS), in agreement with the XPS measurements.

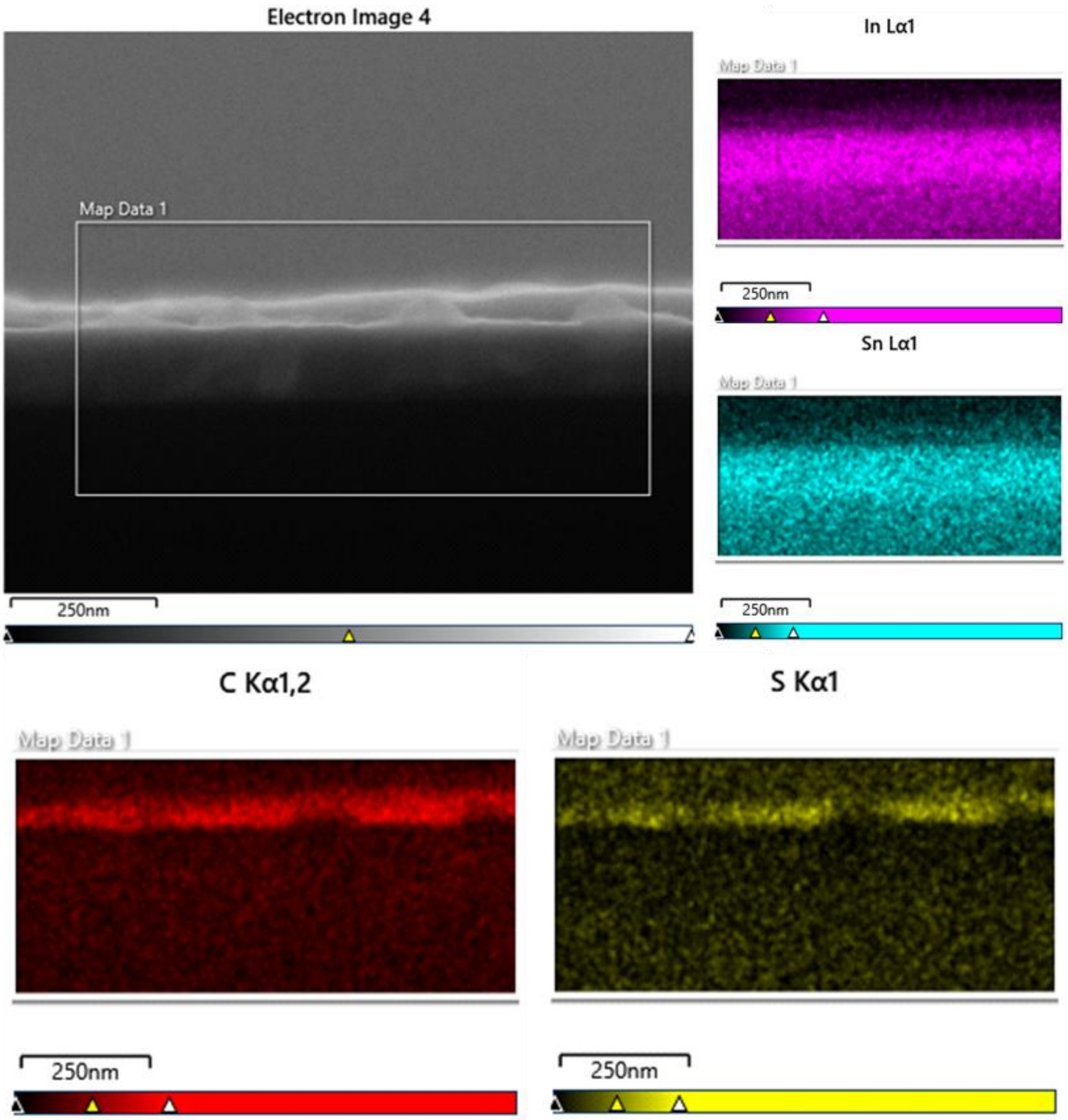

Figure S11 *Cross section image of the CPP-3TB thin film taken by FESEM (up, left) along with the elementary analysis map by EDS of the ITO support (up, right) and the C and S elementary analysis map by EDS of CPP-3TB (down).*

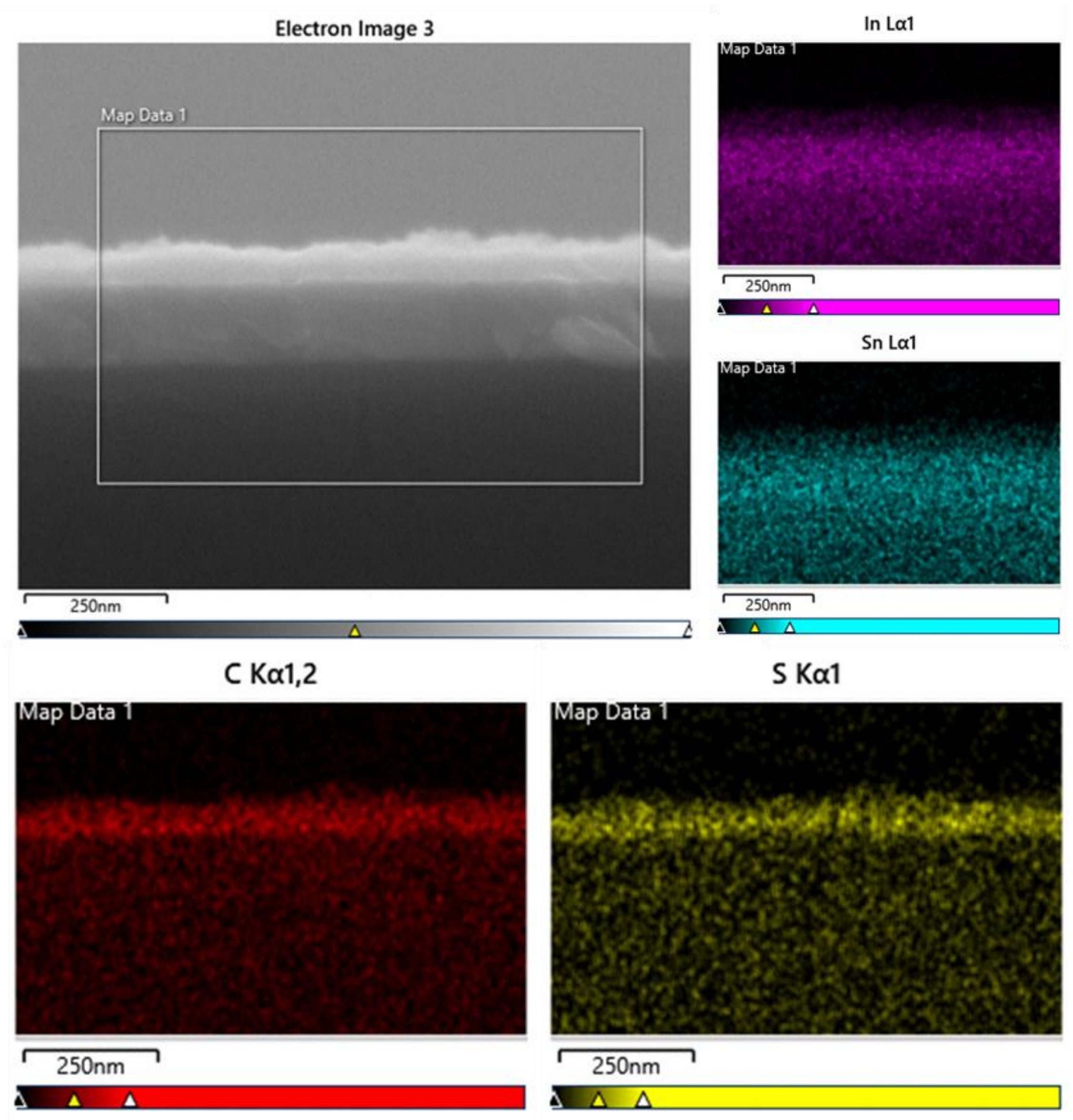

Figure S12. *Cross section image of the IEP-19 thin film taken by FESEM (up, left) along with the elementary analysis map by EDS of the ITO support (up, right) and the C and S elementary analysis map by EDS of IEP-19 (down).*

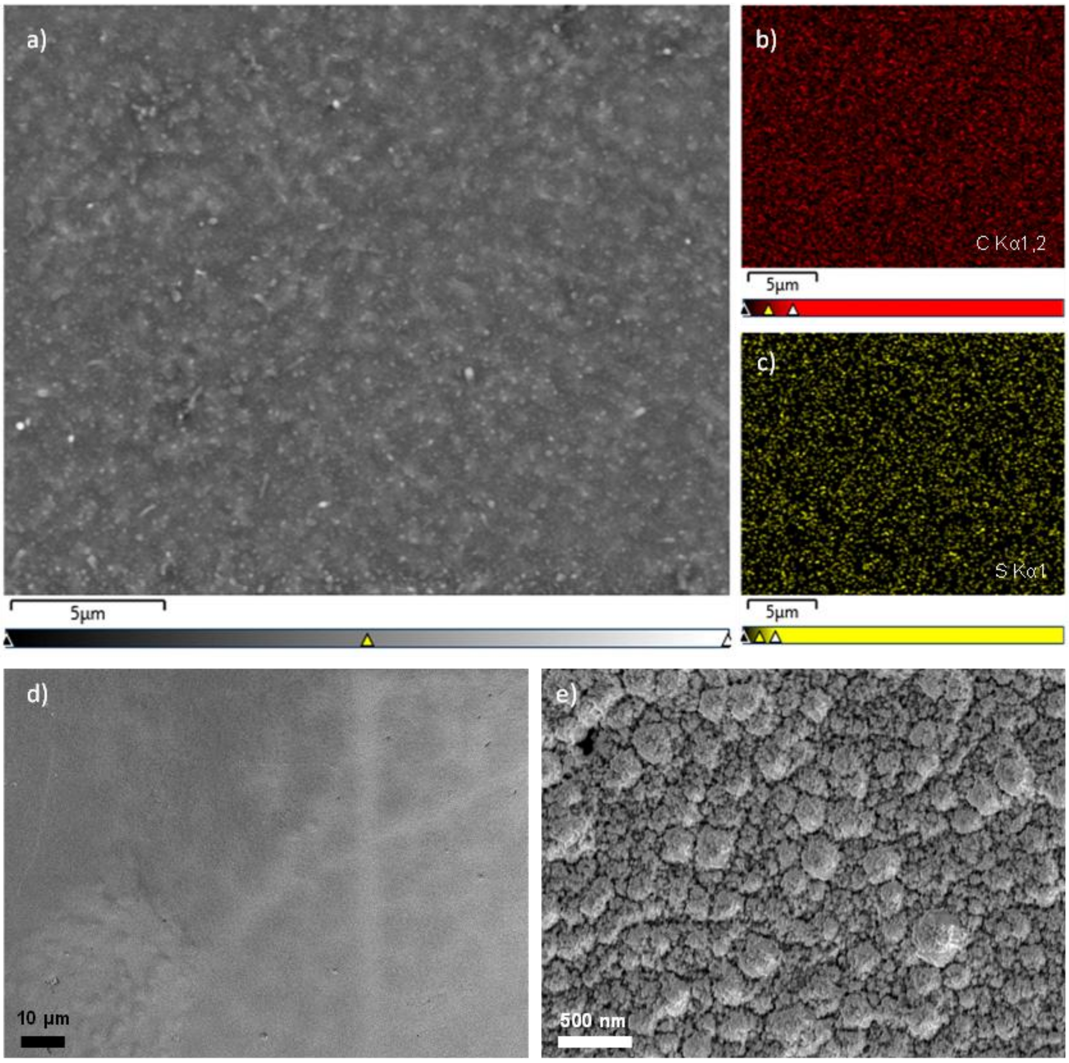

Figure S13. *FESEM images of the CPP-3TB thin film surface, showing its general homogeneity (a and d), along the C (b) and S (c) elementary analysis map by EDS, and its cauliflower-like morphology (e).*

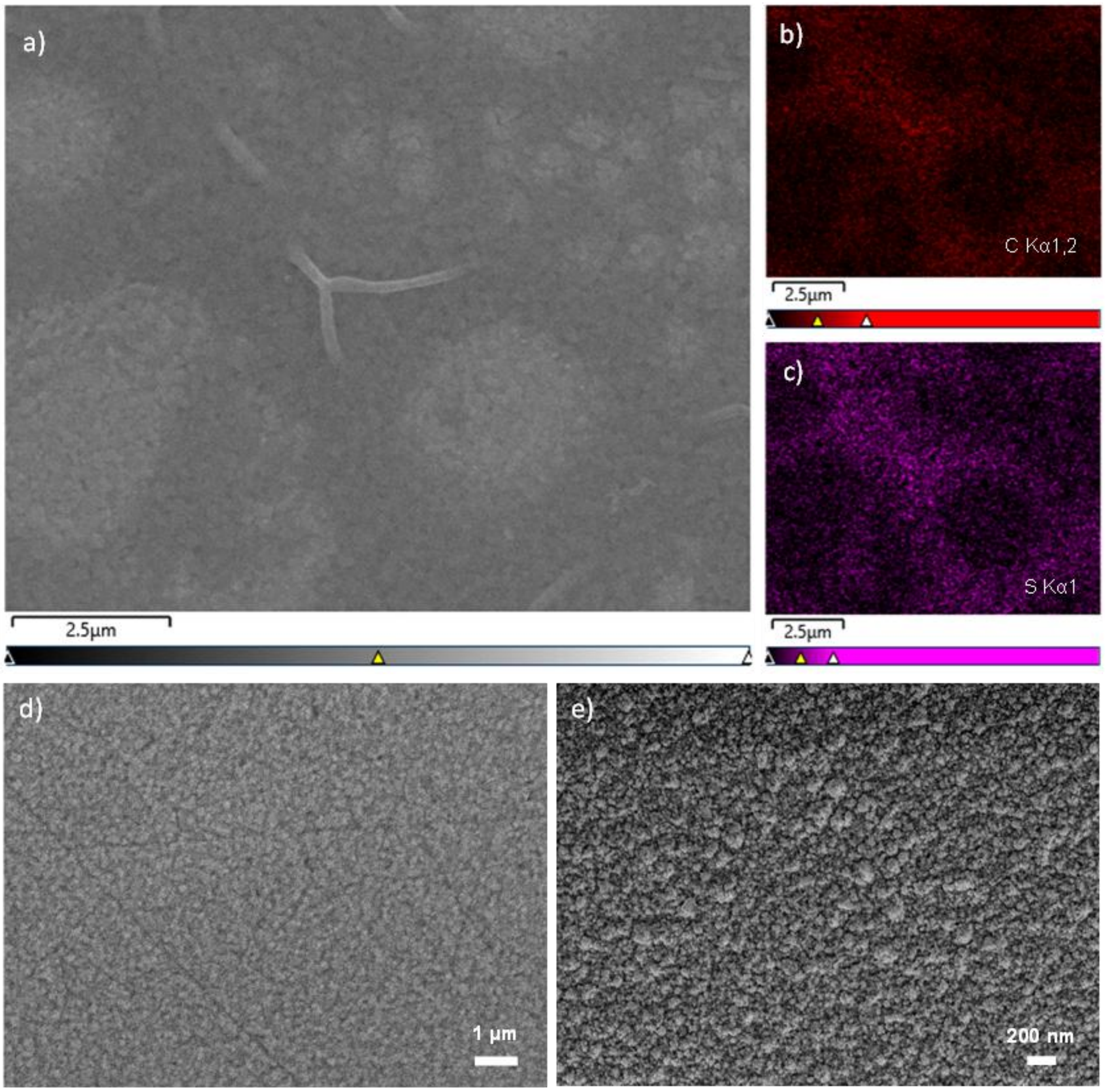

Figure S14. *FESEM images of the IEP-19 thin film surface (a, d and e), along the C (b) and S (c) elementary analysis map by EDS.*

S6. Optoelectronic characterization

Reflectance UV-VIS-NIR spectra were acquired with a Perkin Elmer Lambda 1050 UV-VIS-NIR Spectrometer. The obtained reflectance values (R) were transformed to the Kubelka–Munk function F(R) according to the equation:

$$F(R) = \frac{(1-R)^2}{2R} \qquad equation\ S2$$

The band gap energy was estimated by creating a Tauc plot of (hυF(R))1/n against hυ (where h is Planck's constant, υ is the frequency of vibration and n = 1/2 for direct transitions).

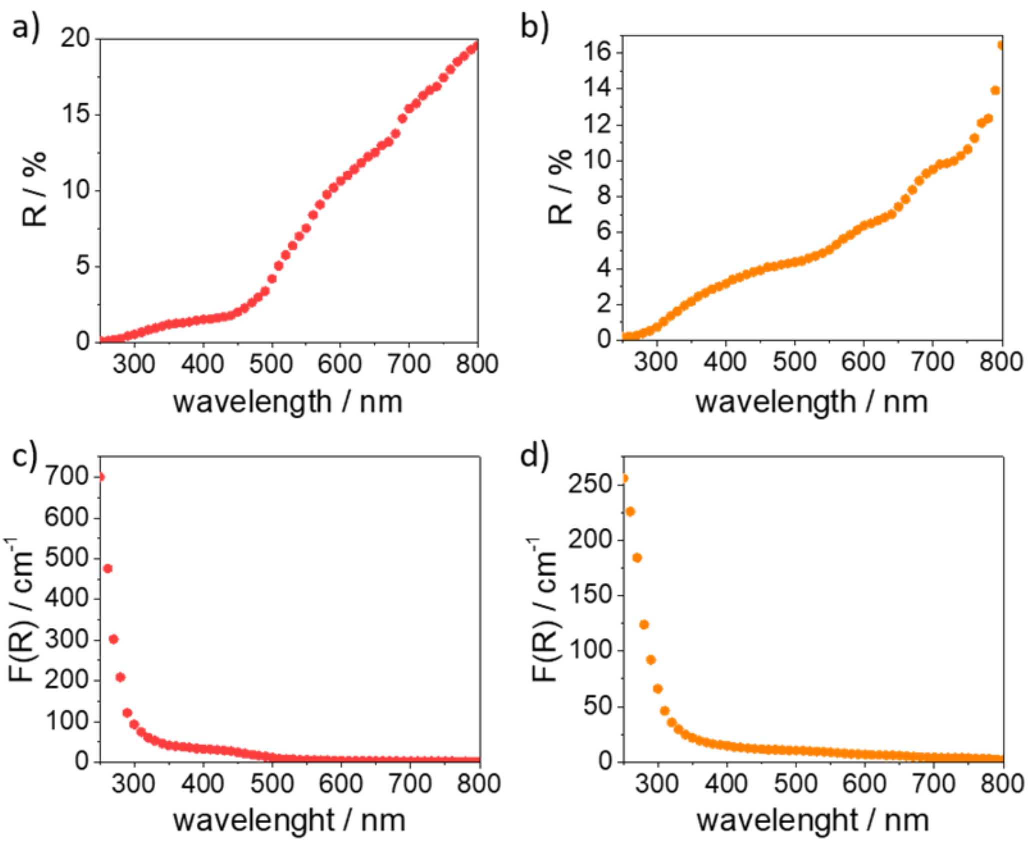

Figure S15. *Reflectance (a, b) and absorption coefficient (c, d) of CPP-3TB (a, c) and IEP-19 (b, d).*

For HOMO and LUMO determination, electrochemical measurements were conducted in acetonitrile solution of tetra-n-butylammonium hexafluorophosphate (0.1 M). A platinum electrode was used as counter electrode and a silver wire (Ag/Ag^+) electrode was used as the pseudo-reference electrode. The CV curves were calibrated using the

ferrocene/ferrocenium (Fc/Fc^+) redox couple as an external standard which was measured under the same conditions before and after the measurement of samples. The LUMO energy levels of monomers and polymers were calculated using the equation:

$$E_{LUMO}(\mathrm{eV}) = \left[4.8 - E_{1/2}(\mathrm{Fc}, \mathrm{Fc}^{+}) + E_{red}, \mathrm{onset})\right] \quad \textit{equation S3}$$

where E_{red}, onset is the onset reduction potential relative to the Ag/Ag^+ reference electrode.[2] The HOMO energy levels of monomers were calculated by subtracting the optical band gap value from the respective LUMO energy levels (EHOMO = ELUMO − Eg, opt). The HOMO energy levels of polymers were estimated by equation:

$$E_{HOMO}(\mathrm{eV}) = -\left[4.8 - E_{1/2}(\mathrm{Fc}, \mathrm{Fc}^{+}) + E_{ox}, \mathrm{onset})\right] \quad \textit{equation S4}$$

where E_{ox}, onset is the onset oxidation potential relative to the Ag/Ag^+ reference electrode.

The half-wave potential of the ferrocene/ferrocenium (Fc/Fc^+) redox couple was estimated from $E_{1/2}(Fc/Fc^{+}) = (E_{ap} + E_{cp})\ /2$, where E_{ap} and E_{cp} are the anodic and cathodic peak potentials, respectively. [10,11]

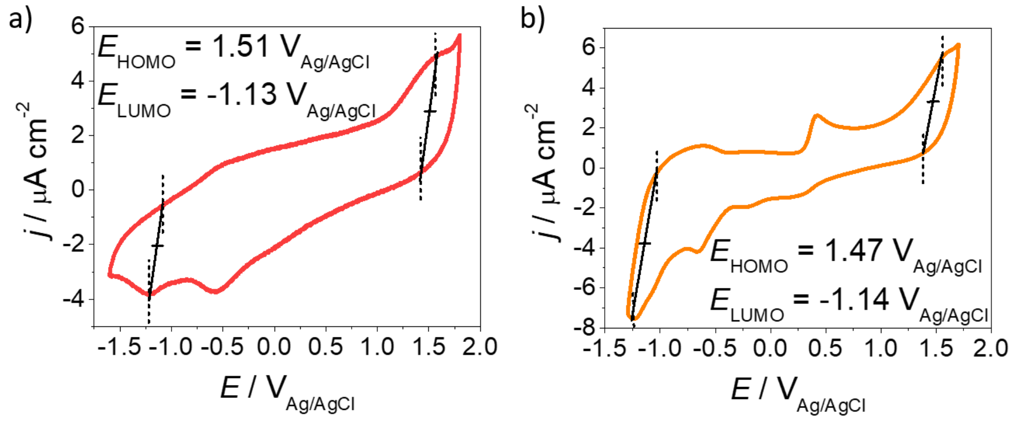

Figure S16. *CV of CPP-3TB (a) and IEP-19 (b) showing the position of their HOMO and LUMO*

S7. (Photo)electrochemical Impedance Spectroscopy (PEIS) of CPPs characterisation

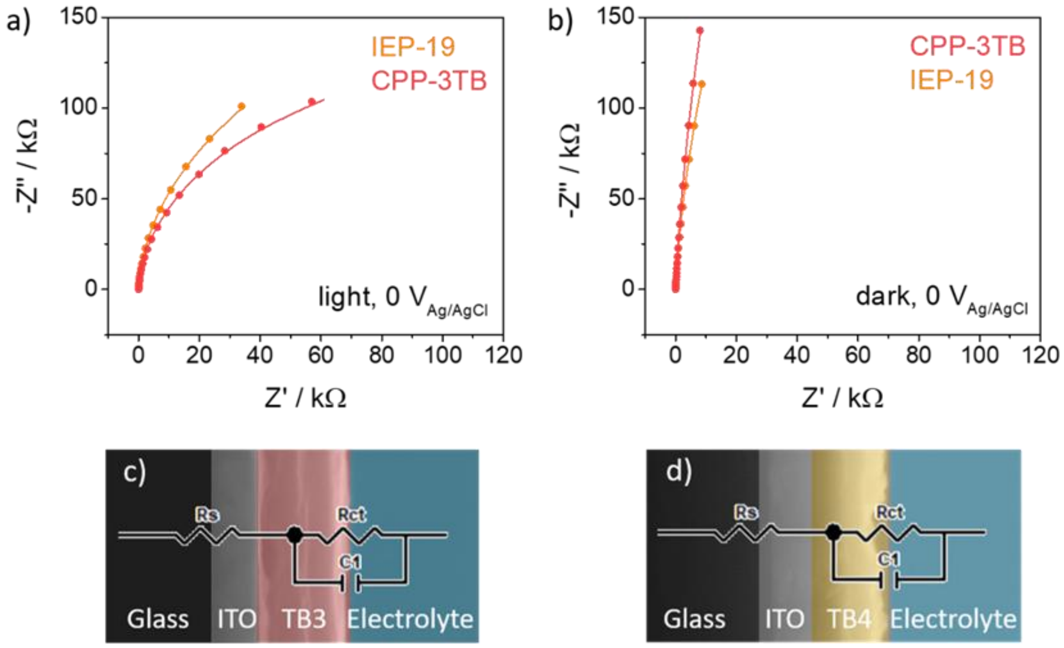

Figure S17*. Nyquist plots of CPP-3TB and IEP-19 under AM1.5G illumination (a) and in the dark (b) at 0 VAg/AgCl in 0.5 M Na_2SO_3. Equivalent Randles circuits for CPP-3TB (c) and IEP-19 (d) thin films.*

S8. Hybrids photoelectrodes preparation and characterisation

<u>TiO_2 synthesis and optimisation</u>

TiO_2 thin films were prepared by a sol-gel method based on the ones by Katoch *et al.*[12] and Conings et al.[13] First, 470 µL of titanium isopropoxide (TIPP) were added dropwise as a precursor to a solution of 101.85 µL of HNO_3 65 v.% as an acid to favour hydrolysis in 2.5 mL of ethanol under mild stirring, which is held for 2 h. Afterwards, 79 µL of deionized water were added to allow hydrolysis, so that the molar ratios of TIPP:ethanol:water:HNO_3 were 1:27:4:1. This mixture was stirred for another hour and then it was diluted with 1-propanol to reach a concentration of 0.127 M. This solution was spun coated on previously cleaned ITO coated glass substrates at 3000 rpm with an acceleration of 1000 rpm/s for 30 s. In order to obtain a crystalline anatase phase, these films were annealed in air at 500 ºC for 1 h. After annealing at 500 °C, the amorphous TiO_2 turned into crystalline anatase. The diffractogram of the obtained

sample can be observed in Figure S18: all the peaks correspond to the anatase phase (PDF number 00-001-0562).

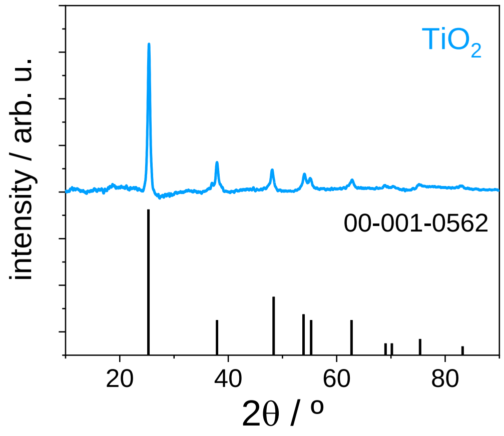

Figure S18. *XRD pattern of an annealed TiO_2 thin film and of anatase (PDF number 00-001-0562).*

A thickness study was carried out to optimise the number of spin-coated layers that would lead to the TiO_2 photoanode with the highest photocurrents. A summary of this study is shown in Figure S19. TiO_2 sol was spin coated on ITO coated glass once, twice, five, ten and twenty times. In order to optimise the optoelectronic properties, the photocurrent, thickness and morphology of each sample were measured by means of LSV, AFM and an optical microscope (Figure S19). Results show an order-of-magnitude increase of both photocurrent and thickness from 1 to 5 layers (Figure S19a and c), with photocurrents of 0.009, 0.063 and 0.551 mA cm^{-2} at 0 $V_{Ag/AgCl}$ and thicknesses of 21.4, 65.5 and 205.4 nm for the 1, 2 and 5-layer samples respectively. Then, as the amount of spin-coated TiO_2 layers increases, there is a sudden drop in the photocurrent of the samples with 10 and 20 layers, with 0.248 and 0.15 mA cm^{-2} respectively.

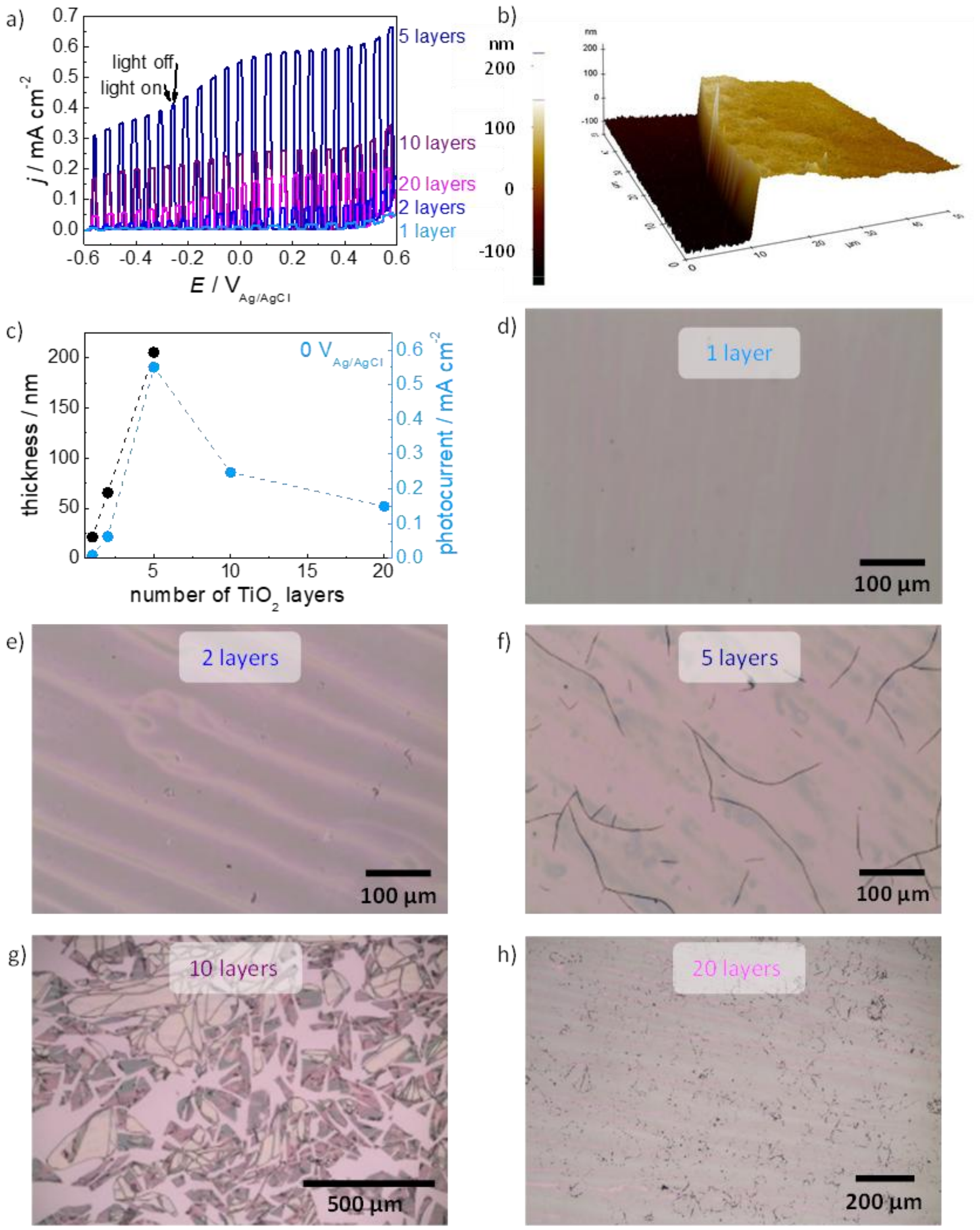

Figure S19. a) LSV with chopped AM 1.5G illumination in 0.5 M Na_2SO_3, b) example of AFM profile, c) relation among the photocurrent, thickness and number of layers and d-h) optic microscope pictures of 1, 2, 5, 10 and 20-layer TiO_2 samples.

This reduction can be explained by looking at the microscope images in Figure S16 d-h: the 10-layer film has collapsed and small pieces of isolated TiO_2 islands replace the conformal thin films seen in the 1- and 2-layer films. Some cracks do already appear in the 5-layer thin film, which does not prevent this sample from achieving the highest photocurrent of all, with 0.55 mA cm^{-2} at 0 $V_{Ag/AgCl}$.

FESEM and EDS Hybrid Photoelectrodes

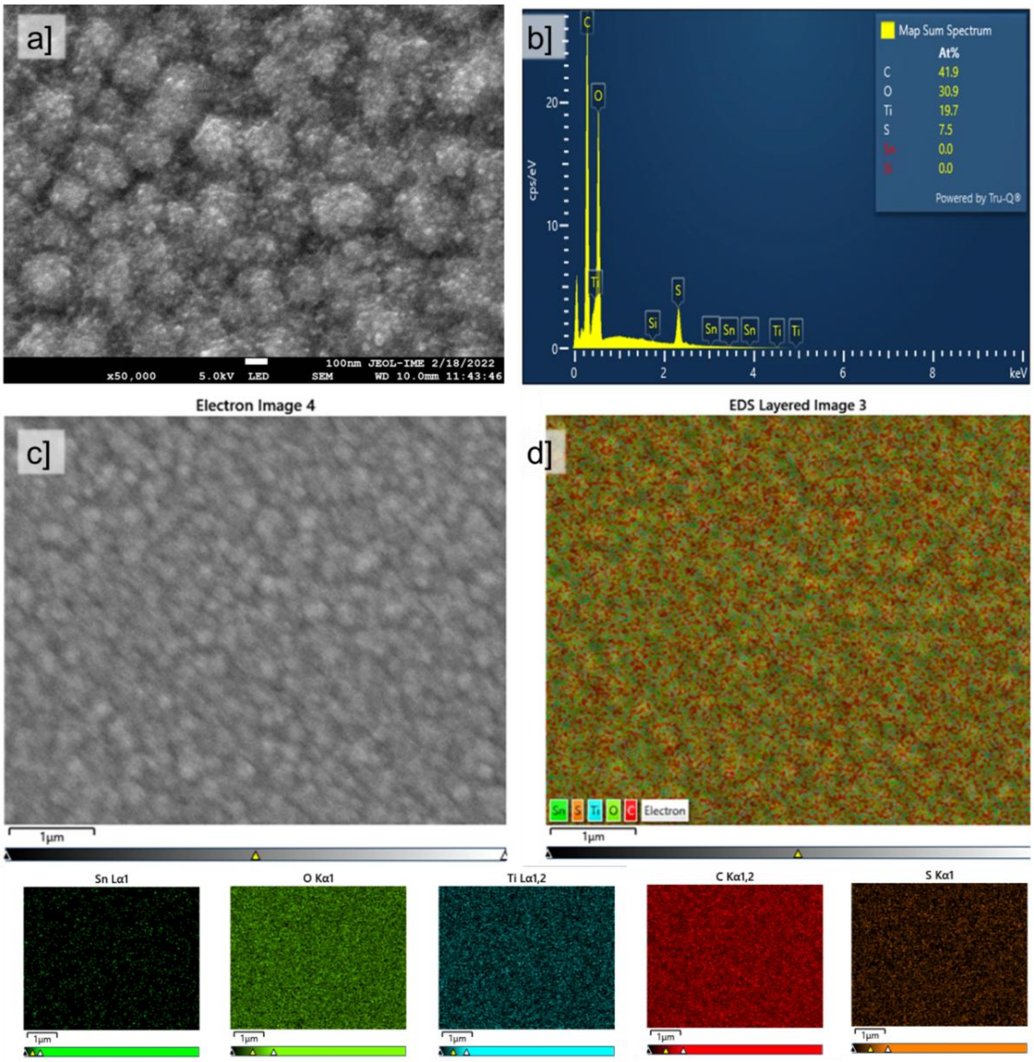

Figure S20. Electron FESEM images and EDS maps of CPP-3TB@TiO_2 photoanode.

Incident Photon-to-Current Efficiency

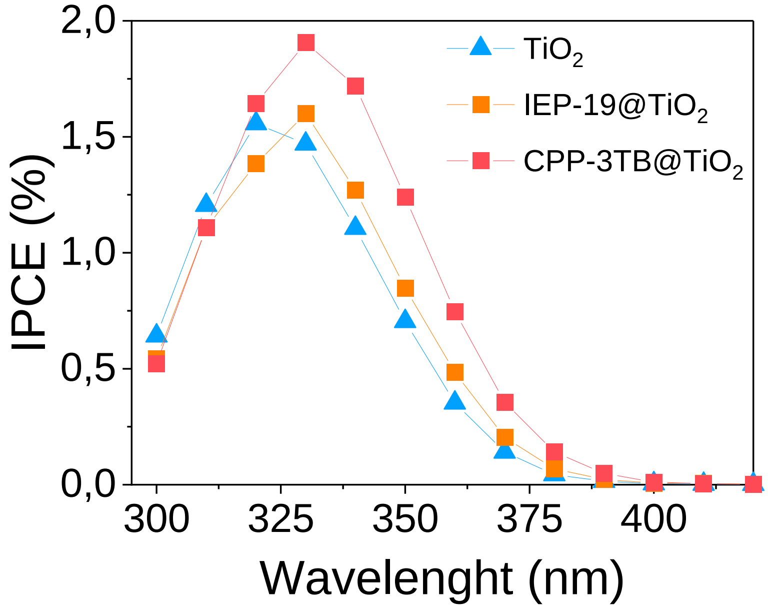

Figure S21. Incident Photon-to-Current Efficiency (IPCE) of CPP-3TB/TiO_2 (a) and IEP-19/TiO_2 (b) photoanodes compared to bare TiO_2 in 0.5 M Na_2SO_3

S9. Transient Absorption Spectroscopy (TAS) characterisation

TAS measurements were carried out with a LP980 equipment from Edinburgh Instruments (LP980) based on an optical parametric oscillator (OPO) pumped by the third harmonic of a Nd:YAG laser (EKSPLA). The selected excitation wavelength for monitoring the signals was 355 nm with single low energy pulses of 300 mJ/pulse of ca. 5 ns duration to avoid the organic degradation of material while a pulsed xenon flash lamp (150 W) was employed as probe source. The probe light is dispersed through a monochromator (TMS302-A, grating 150 lines/mm) after it has passed the sample and then reaches a PMT detector (Hamamatsu Photonics) to obtain the temporal profile. The thin-films of Indium Tin Oxide (ITO) containing the photoactive material (TiO_2, conjugated porous polymer or hybrid) were immersed in H_2O in 10 x 10 mm^2 cuvettes with four clear sides and deaerated for 15 min with N_2 before acquisition. All transient signals were recorded at room temperature.

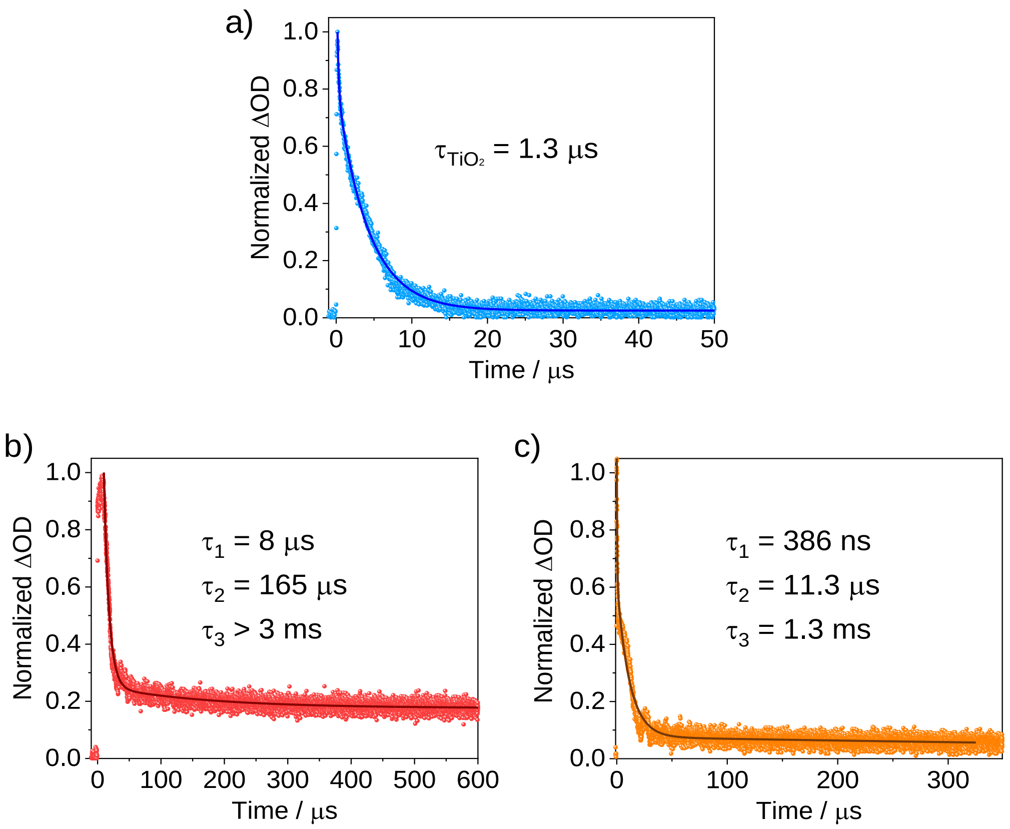

Figure S22. Normalized decays traces and fitting for a) TiO_2, b) CPP-3TB/TiO_2 and c) IEP-19/TiO_2.

References

[1] "Stille Coupling," can be found under https://www.organic-chemistry.org/namedreactions/stille-coupling.shtm, **n.d.**

[2] J. L. Brusso, O. D. Hirst, A. Dadvand, S. Ganesan, F. Cicoira, C. M. Robertsons, R. T. Oakley, F. Rosei, D. F. Perepichka, *Chemistry of Materials* **2008**, *20*, 2484.

[3] M. Barawi, L. Collado, M. Gomez-Mendoza, F. E. Oropeza, M. Liras, V. A. de la Peña O'Shea, *Advanced Energy Materials* **2021**, *2101530*, 1.

[4] A. Palma-Cando, G. Brunklaus, U. Scherf, *Macromolecules* **2015**, *48*, 6816.

[5] C. Gu, Y. Chen, Z. Zhang, S. Xue, S. Sun, K. Zhang, C. Zhong, H. Zhang, Y. Pan, Y. Lv, Y. Yang, F. Li, S. Zhang, F. Huang, Y. Ma, *Advanced Materials* **2013**, *25*, 3443.

[6] F. Li, H. Yang, W. Li, L. Sun, *Joule* **2018**, *2*, 36.

[7] R. B. Ambade, S. B. Ambade, N. K. Shrestha, R. R. Salunkhe, W. Lee, S. S. Bagde, J. H. Kim, F. J. Stadler, Y. Yamauchi, S. H. Lee, *Journal of Materials Chemistry A* **2017**, *5*, 172.

[8] C. Gu, N. Huang, Y. Chen, L. Qin, H. Xu, S. Zhang, F. Li, Y. Ma, D. Jiang, *Angew. Chem. Int.* **2015**, *54*, 13594.

[9] D. García Heredia, Síntesis de Polímeros Conjugados Porosos Para Fotogeneración de Hidrógeno Por Procesos Fotoelectroquímicos, Universidad Complutense de Madrid, **2019**.

[10] J. Pommerehne, H. Vestweber, W. Guss, R. F. Mahrt, H. Bässler, M. Porsch, J. Daub, *Advanced Materials* **1995**, *7*, 551.

[11] G. Gritzner, *Pure Appl. Chem.* **1990**, *62*, 1839.

[12] A. Katoch, H. Kim, T. Hwang, S. S. Kim, *Journal of Sol-Gel Science and Technology* **2012**, *61*, 77.

[13] B. Conings, L. Baeten, T. Jacobs, R. Dera, J. D'Haen, J. Manca, H. G. Boyen, *APL Materials* **2014**, *2*, DOI 10.1063/1.4890245.
